# MicroRNAs in Parkinson’s disease: a systematic review and diagnostic accuracy meta-analysis

**DOI:** 10.1038/s41598-023-43096-9

**Published:** 2023-09-28

**Authors:** Diane Guévremont, Joyeeta Roy, Nicholas J. Cutfield, Joanna M. Williams

**Affiliations:** 1 Department of Anatomy, University of Otago, Dunedin, New Zealand; 2 Brain Health Research Centre, Dunedin, New Zealand; 3 Department of Medicine, University of Otago, Dunedin, New Zealand

**Keywords:** Parkinson's disease, Biomarkers

## Abstract

Current clinical tests for Parkinson’s disease (PD) provide insufficient diagnostic accuracy leading to an urgent need for improved diagnostic biomarkers. As microRNAs (miRNAs) are promising biomarkers of various diseases, including PD, this systematic review and meta-analysis aimed to assess the diagnostic accuracy of biofluid miRNAs in PD. All studies reporting data on miRNAs expression in PD patients compared to controls were included. Gene targets and significant pathways associated with miRNAs expressed in more than 3 biofluid studies with the same direction of change were analyzed using target prediction and enrichment analysis. A bivariate model was used to calculate sensitivity, specificity, likelihood ratios, and diagnostic odds ratio. While miR-24-3p and miR-214-3p were the most reported miRNA (7 each), miR-331-5p was found to be consistently up regulated in 4 different biofluids. Importantly, miR-19b-3p, miR-24-3p, miR-146a-5p, and miR-221-3p were reported in multiple studies without conflicting directions of change in serum and bioinformatic analysis found the targets of these miRNAs to be associated with pathways important in PD pathology. Of the 102 studies from the systematic review, 15 studies reported sensitivity and specificity data on combinations of miRNAs and were pooled for meta-analysis. Studies (17) reporting sensitivity and specificity data on single microRNA were pooled in a separate meta-analysis. Meta-analysis of the combinations of miRNAs (15 studies) showed that biofluid miRNAs can discriminate between PD patients and controls with good diagnostic accuracy (sensitivity = 0.82, 95% CI 0.76–0.87; specificity = 0.80, 95% CI 0.74–0.84; AUC = 0.87, 95% CI 0.83–0.89). However, we found multiple studies included more males with PD than any other group therefore possibly introducing a sex-related selection bias. Overall, our study captures key miRNAs which may represent a point of focus for future studies and the development of diagnostic panels whilst also highlighting the importance of appropriate study design to develop representative biomarker panels for the diagnosis of PD.

## Introduction

Parkinson’s disease (PD) is characterized by the progressive loss of dopaminergic neurons in the substantia nigra (SN)^1^. Clinically, diagnosis relies on the presentation of the cardinal motor features of PD, bradykinesia, rigidity, and tremor. The onset of these motor symptoms is generally consistent with a loss of approximately 60% of the SN dopaminergic neurons, along with widespread neural network disruption^2^. Diagnosis can be aided by ancillary dopamine imaging tests, however, these are cost-prohibitive and not widely accessible. Therefore, to date, the gold standard for PD diagnosis remains neuropathology. However, early diagnosis of PD is the key to developing and testing novel therapies at a disease stage where they might have the greatest impact on limiting neuronal death and therefore improving the quality of life of patients. Thus, there is an urgent need for accessible peripheral markers capable of reflecting the central nervous system (CNS) pathology underlying PD.

MicroRNAs (miRNAs) are small non-coding RNAs which participate in post-transcriptional control of gene expression^3^. They play a regulatory role in numerous biological processes including the cell cycle, apoptosis, and stress response in cells and have been shown to be dysregulated in postmortem brain tissue derived from individuals with PD^4,5^. Importantly, miRNAs are released from the brain and are found in various biofluids including blood, plasma, serum, peripheral blood mononuclear cells (PBMCs), cerebrospinal fluid (CSF) and extracellular vesicles such as exosomes^6–10^. As they are also remarkably stable in biofluids^11^, assessing biofluid miRNA levels may act as an effective biomarker of PD and be useful not only in diagnosis, but also in monitoring disease progression and reflect responses to therapy.

To date, there is a distinct lack of consensus on which miRNA or combinations of miRNAs are the most effective diagnostic test for PD. This may be due to methodological differences in preclinical blood processing, method of miRNA analysis, heterogeneity in PD pathology, or small sample size and potential biases introduced by limitations in control groups. Moreover, identification of PD-specific miRNAs, rather than ones affected by neurodegeneration in general, is also challenging. Nevertheless, significant efforts are being made into identifying signatures of miRNAs capable of differentiating between PD patients and controls. For example, Schulz et al. identified 13 differentially expressed miRNAs in the brain and blood^12^. The importance of each miRNA in this study was based on the significance of *p* values which were generated from effect size meta-analyses conducted on each miRNA. Diagnostic accuracy meta-analyses have been published on miRNAs but in other CNS disorders such as Alzheimer’s disease and multiple sclerosis^13,14^, yet to date, only 1 study^15^ has assessed the diagnostic accuracy of biofluid-derived miRNAs in PD patients. The importance of diagnostic accuracy meta-analyses lies in their ability to provide information on the area under the curve (AUC), sensitivity and specificity, which are among the determining factors of clinical utility of miRNAs as an evidence-based diagnostic tool for PD. Here, we performed a systematic review to update and amalgamate all current knowledge on miRNAs from brain tissue and biofluids and to identify miRNAs which are consistently reported to be changed in PD patients compared to controls. Using meta-analysis, we evaluated the diagnostic utility of miRNA from biofluids capable of discriminating between PD patients and controls.

## Materials and methods

### Search strategy

This study protocol was registered in PROSPERO (CRD42018104269) and was conducted according to the Preferred Reporting Items for Systematic Reviews and Meta-Analyses (PRISMA) guidelines^16^. A literature search was performed on several databases including PubMed, Scopus, Web of Science, and the Cochrane Library, through to July 2022, using *microRNA, miRNA, biomarkers, Parkinson’s disease*, and *neurodegeneration* as search terms. The PubMed advanced search strategy was as follows: *((((((((microRNAs[Title/Abstract]) OR microRNA[Title/Abstract]) OR miRNA[Title/Abstract]) OR miRNAs[Title/Abstract]) OR MIR[Title/Abstract]) OR biomarkers[Title/Abstract]) OR biomarker[Title/Abstract])) AND ((((Parkinson’s disease[Title/Abstract]) OR Parkinson’s[Title/Abstract]) OR neurodegenerative[Title/Abstract]) OR neurodegeneration[Title/Abstract])*. This search strategy was individually adapted for each database. Only studies in English were assessed for inclusion. The references of all relevant studies were searched to identify additional eligible studies. The title, abstract and/or full texts of studies were used, as needed, to gauge eligibility.

### Study selection

All original studies, conference posters and abstracts reporting data on miRNA expression in PD patients and healthy controls (HCs) were included in the systematic review. All studies on PD patients, irrespective of treatment status and type, were included. Studies were included in the meta-analysis if they reported data on: (1) patients diagnosed with PD (clinically/neuropathologically), (2) miRNA expressions in biofluids of PD patients and controls, (3) samples size, sensitivity, and specificity of individual or combinations of miRNAs, and (4) in case of multiple individual miRNA results within the same study (multiple individual AUC, one for each miRNA), only the top miRNA data entry with the highest AUC was retained for further analysis in order to not introduce statistical biases.

Exclusion criteria included data which were: (1) irrelevant, comparing miRNA expressions from PD with other neurodegenerative diseases only without reporting data on controls, (2) on miRNA expressions from non-human subjects, (3) overlapping with other included studies, and (4) on miRNA expression analysis based on non-experimental techniques, such as computational analyses.

### Data extraction

The following patient data were extracted: first author, year of publication, country of origin, sample size, number of males in patient and control cohorts, mean ages of patients and controls, disease duration, type of PD patients (sporadic, familial or mixed PD), specific genetic mutations in familial PD patients, clinical phenotype of PD patients, Hoehn and Yahr (H&Y) stage, PD medication status, type and dose, levodopa equivalent daily dose (LEDD), diagnostic criteria used to make a clinical diagnosis of PD and Braak stages of neuropathologically-confirmed PD patients.

The following miRNA data were extracted: sample type including specific brain regions used in postmortem studies, experimental method of miRNA analysis and normalization, miRNA expression and direction of change, and diagnostic accuracy indices (sensitivity, specificity and AUC) if available. Wherever possible, designations of -3p and -5p were added to miRNAs (if not reported by the original study) using miRbase (www.mirbase.org, Version 22.1), websites of the manufacturers of specific miRNA primers used in the original study, or other publications cited in by the original study on the miRNA in question^17^. Despite this, if it was not possible to assign -3p or -5p to specific miRNAs, no assumptions were made on designation. These miRNAs were, therefore, not included in tallies of their -3p/-5p counterparts in the systematic review.

### Quality assessment

Methodological quality of every included study was evaluated using the QUADAS-2 (Quality Assessment of Diagnostic Accuracy Studies-2) quality assessment tool, which uses signaling questions to assess risk of bias in four domains (patient selection, index test, reference standard, and flow and timing) and applicability in three domains (patient selection, index test, and reference standard)^18^. Quality was assessed by JR, JW, and DG who independently assigned each domain with scores of low, medium, or high after evaluating methodological quality of every included study. Any disagreements between the three reviewers were resolved by consensus after discussion.

### Bioinformatic analysis

The gene targets of the overlapping miRNAs from the systematic review were identified using the DIANA mirPathv3-TarBase v7.0 (https://dianalab.e-ce.uth.gr/html/mirpathv3/index.php?r=mirpath) and the miRTarBase version 8.0 (https://mirtarbase.cuhk.edu.cn/) databases^19,20^. All stringency levels were set to *p* < 0.05 and the “genes union” analysis method was used to identify all gene targets of selected miRNAs from the *Homo sapiens* species. Enrichment analyses were conducted on these gene targets using the enrichment tool Enrichr (http://amp.pharm.mssm.edu/Enrich) (Wikipathways database), which enables visualization of miRNA gene targets and the biological pathways with which they are interacting^21,22^.

### Statistical analysis

Statistical analyses were performed using the MIDAS and METANDI modules on STATA version 17 (StataCorp 2021, College Station, TX, USA)^23^. All miRNAs reported to be changed between PD patients and controls were extracted. The construction of 2 × 2 contingency tables based on sensitivity, specificity and the number of patients and controls from each study allowed for the calculation of true positives (TP) or individuals with PD who test positive for the disease, false positives (FP) or individuals without PD who test positive for the disease, true negatives (TN) or individuals without PD who test negative for the disease and false negatives (FN) or individuals with PD who test negative for the disease. Sensitivity was calculated as TP divided by the sum of TP and FN and indicates how well the test can detect positive cases. Specificity was calculated as TN divided by the sum of TN and FP and indicates how well the test correctly identified negative cases.

The bivariate mixed-effects binomial regression model in the MIDAS module calculates the sensitivity and specificity and the relationship between them, while considering both within- and between-study variability^24^. The following pooled diagnostic accuracy indices with their 95% confidence intervals (CI) were calculated: sensitivity, specificity, positive likelihood ratio (PLR), negative likelihood ratio (NLR), diagnostic score and diagnostic odds ratio (DOR). Likelihood ratios (LR) assess the value of performing a diagnostic test. The PLR (sensitivity divided by 1—specificity) indicates how much more likely a positive result is likely to occur in individuals with PD compared to those without. The NLR (1—sensitivity divided by specificity) indicates how much less likely a negative result is likely to occur in individuals with PD to those without. The diagnostic score measures the accuracy of a test in distinguishing between different conditions and is useful in evaluating how well a test can correctly identify or exclude a particular condition. The DOR is a measure of the odds of a positive test in those with disease relative to the odds of a positive test in those without disease^25^. Additionally, the positive-predictive value (PPV), the proportion of patients with positive test results who are correctly diagnosed, (TP divided but the sum of TP and FP), and the negative predictive value (NPV), the proportion of patients with negative test results who are correctly diagnosed (TN divided by the sum of TN and FP), were also calculated^25^.

Overall diagnostic accuracy was assessed using summary receiver operating characteristic (SROC) curves and associated AUC values. The SROC curve represents the relationship between the TP and FP rates across all studies while considering differing cut-off thresholds of included studies. Clinical utility analysis was conducted using the Fagan’s nomogram which graphically displays the pre-test probability (probability of the screened patient having PD) and the post-test probability (probability of the patient having PD after obtaining the results of the miRNA-based diagnostic test), which are based on the likelihood ratios of miRNAs used to differentiate patients and controls^26–28^.

Heterogeneity was assessed using Cochran’s Q statistic and the I2 test; *p* value < 0.05 (Q statistic) and/or I2 > 50% was considered statistically significant. The Spearman correlation coefficient was used to identify potential threshold effects caused by varying diagnostic thresholds for sensitivities and specificities between studies. Sources of heterogeneity were further explored using meta-regression and subgroup analyses based on study characteristics sample type (serum vs plasma, CSF, PBLs, PBMCs, saliva and whole blood), origin (China vs rest of the world), sex (higher number of men vs women in each group), platform used for miRNA quantification (TaqMan vs SYBR Green), normalization used in miRNA expression analysis (stable miRNAs vs U6 small nuclear RNA and other methods).

A Friedman test was used to determine whether or not there was a statistically significant difference between the means of all 4 groups studied (sample size = PD men, PD women, Control men and Control women). This test was used because we had determined that the data was not normally distributed. A p-value < 0.05 indicated that the group medians were not equal and therefore there was a significant difference. This was followed by Dunn’s post hoc tests to confirm where the differences occurred between these groups.

Publication bias analysis was performed using the Deeks’ linear regression of log odds ratios against 1/square root of the effective sample size to test for asymmetry of funnel plots, with *p* < 0.10 for the slope indicating significant asymmetry with a high likelihood of publication bias.

## Results

### Systematic review

**Figure 1:**
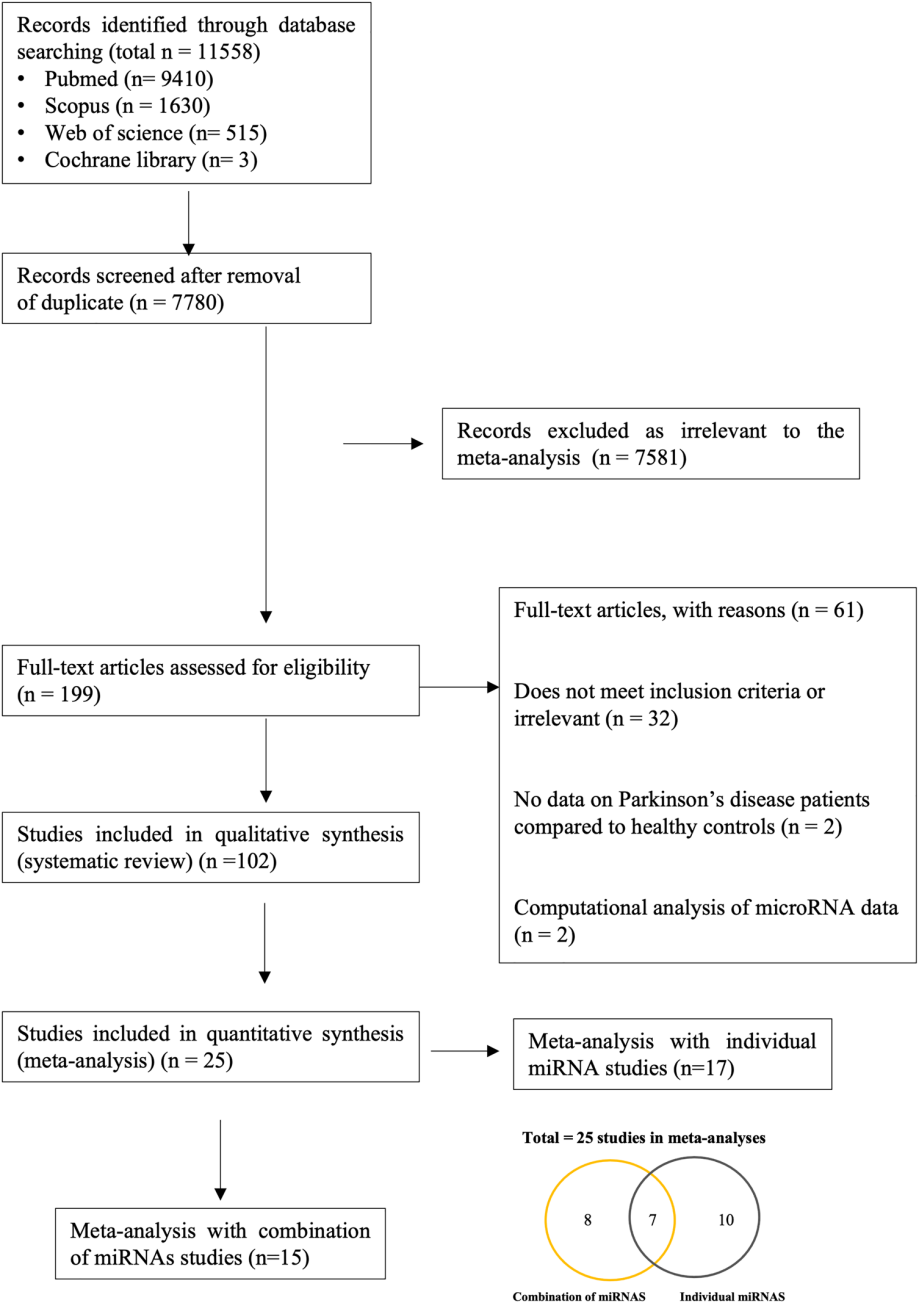
The PRISMA flow chart showing study identification and inclusion and exclusion criteria for all studies included in the systematic review and meta-analyses.

The PRISMA flow diagram detailing study identification, inclusion and exclusion are presented in Fig. 1. A total of 11,558 studies were identified through database searching including 16 studies added by manually searching through the references of relevant studies. The titles and abstracts of all records were then screened for relevance and the full texts of 199 studies were resultantly assessed for eligibility according to the study selection criteria (refer Methods). This number was further filtered by for example review articles, insufficient diagnostic criteria provided by the authors or lack of control information. Finally, 102 studies were included in the systematic review (Supplementary Table S1 and S2). From these, 25 studies were included for meta-analyses (Table 1). Of the 25 studies, 15 provided sensitivity and specificity data on combinations of miRNAs (Table 1a)^29–44^ and were pooled for a diagnostic meta-analysis (Fig. 3, 4, 5a; Table 3, Supplementary Figure S2-S3). Of the 15 studies, 7 also provided diagnostic accuracy data on individual miRNAs in addition to miRNA combinations^29,31–33,36,37,43^. A further 10 studies only provided sensitivity and specificity data on individual miRNAs^45–54^. The individual miRNA studies (17 in total; Table 1b) were pooled in a separate diagnostic subgroup meta-analysis. In this article, we have focused on the data generated from the studies using combinations of miRNAs since these outperformed diagnostic data from when the individual miRNAs were pooled and meta-analysed.

**Table 1:**
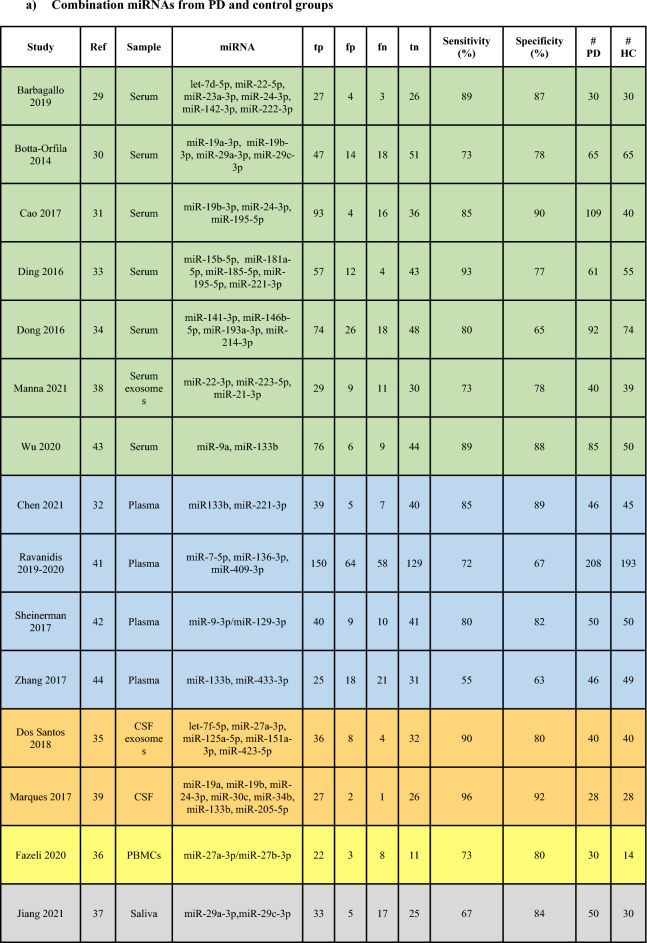

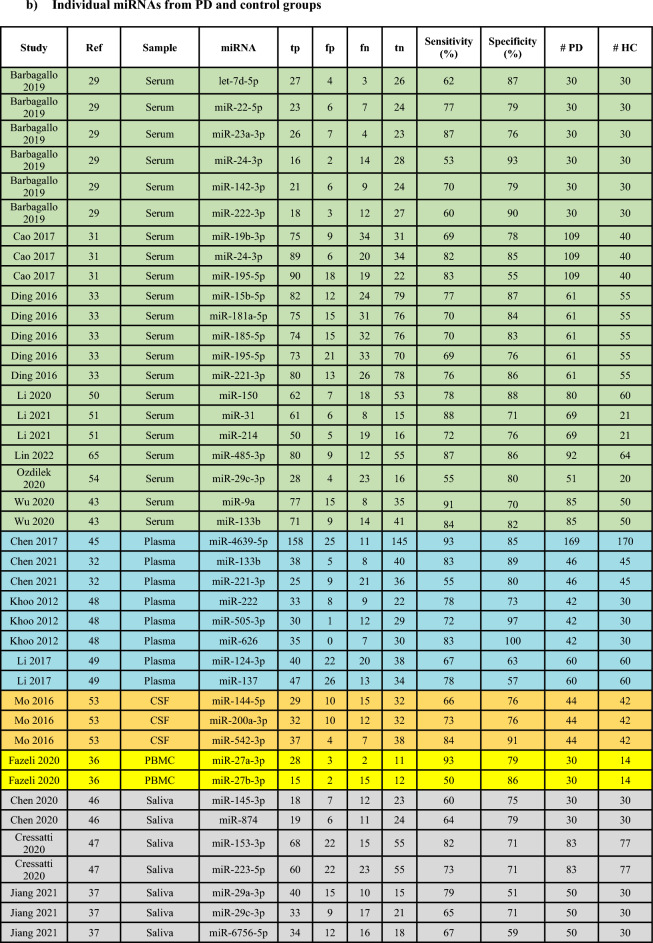
Biofluid studies reporting diagnostic accuracy data on (a) combination and (b) individual miRNAs from PD and control groups.

Green: Serum; Blue: Plasma; Orange: CSF; Yellow: PBMCs; Grey: Saliva; tp: true positives, fp: false positives, fn: false negatives, tn: true negatives, PD: PD patients, HC: controls.

Data on miR-1826 reported by Khoo et al. were excluded from this meta-analysis as it has now been reported to be a fragment of 5.8S ribosomal RNA (rRNA)^17,48^. Chatterjee et al. performed computational analyses using data from Hoss et al. and Soreq et al. and was therefore excluded^4,55,56^. Wake et al. identified novel miRNAs in tissue from the prefrontal cortices of PD patients and controls, whereas a complementary study by Hoss et al. reported data on differentially expressed known miRNAs in the same data^4,57^. Therefore, data from both these studies were reported. Ravanidis et al*.* pooled results from two studies^40,41^, therefore only their pooled results analysis was included in this systematic review, and meta-analysis^41^.

The characteristics of all included studies are presented in Supplementary Table S1 which includes all patient and control demographic data and Supplementary Table S2 which presents all data regarding the source, method of analysis and direction of change of miRNAs reported to be changed between PD patients and controls. The QUADAS-2 tool was used to assess the quality of all included studies (Supplementary Figure S1). No study was assessed as exhibiting an overall high risk of bias.

The most common country of origin of included studies was China (n = 42)^9,10,31–34,37,43–46,49–51,53,58–84^ followed by the USA (n = 13)^4,42,48,57,85–93^, Spain (n = 10)^30,94–102^, Italy (n = 10)^29,38,103–110^, Germany (n = 6)^35,111–114^, Iran (n = 5)^36,115–118^, Japan (n = 2)^119,120^, Portugal (n = 2)^121,122^ and Turkey (n = 2)^54,123^. Only 1 study originated from each of the following countries: Brazil^8^, Canada^47^, Denmark^125^, Egypt^128^, Netherlands^39^, Norway/Sweden^91^, Romania^132^, Russia^124^, Taiwan^126^, and the United Kingdom^127^. The sample sizes of included studies ranged from 6 to 59. The mean ages of PD patients ranged from 29.4 to 83 years with mean disease durations ranging from 1.4–16.2 year. The mean ages of controls ranged from 31.2–85 years.

The most frequent etiology of PD reported was idiopathic/sporadic (n = 29 studies)^30,37,39,41,44,45,48,49,58,62,64,68,70,77–79,86,88,94,96,101,110–112,121,122,126,128,129^ PD patients were commonly receiving medical and/or surgical antiparkinsonian treatment at time of inclusion (n = 35 studies)^8,29,37,38,43,45,47,48,51,54,56,58,63,77–80,83,90,99,100,103–107,109,110,122–125,127,129,132^. Of these, Soreq et al. included patients who were on PD medications and subthalamic nucleus deep brain stimulation (STN-DBS0^56^. A total of 14 studies reported the mean LEDD of their PD patients, which ranged from 197 to 939 mg/day^37,43,45,47,54,58,63,100,103,104,107,109,124,125^. Of these, several studies also included subsets of patients who were untreated (n = 12 studies)^8,48,51,58,77,78,99,105,106,110,124,132^, whereas 6 studies included only untreated PD patients^60,75,83,91,95,99^. Moreover, few studies reported the clinical phenotype of PD in their patient cohort. Of these, Jin et al. and Zhao et al. included PD patients with tremor-dominant, bradykinesia and rigidity-dominant and mixed clinical phenotypes,^10,82^ Dos Santos et al. included only the akinetic-rigid phenotype,^35^ and Alieva et al. included patients with the mixed clinical phenotype^124^.

The Hoehn and Yahr Scale (H&Y) denoting disease severity in PD patients was not reported by over half the studies included in this systematic review. This scale ranges from Stage 0 to 5 with higher stages suggesting increased disability in PD patients. Stages ≤ 2 indicate mild unilateral PD whereas stages ≥ 3 correlate with bilateral mild to moderate PD. Advanced PD with severe disability is represented in Stages 4 and 5^130^. Of the studies which reported these data, the H&Y stage of included patients ranged from 1 to 5 in 12 studies,^30,31,33,34,44,47,49,51,66,81,82,103^ 1–4 in 8 studies,^38,48,74,105,112,115–117^ 1–3 in 17 studies,^8,10,29,32,36,45,50,58,69,71,75,78,100,107,121,122,132^ and was reported as being < 2.5 in 11 studies^35,37,39,46,53,54,63,70,91,124,125^. Further, over a quarter of the included studies did not specify the diagnostic criteria used to make a clinical diagnosis of PD in their included patients. Of the studies which did report these data, the United Kingdom Parkinson’s Disease Society Brain Bank Criteria (UKPDSBBC) was the most frequently used diagnostic criteria (n = 48 studies)^8,10,30,31,35,39,44,45,47–51,53,54,58–60,62,64–68,70,75,80–82,85,86,89,91,95,98,104,107–110,112,117,118,121,124,125,128,129^. Other clinical criteria used were the Gelb (n = 5 studies),^29,103,105,106,126^ the Queen Square Brain Bank (n = 2 studies),^42,94^ and the Movement Disorders Society (MDS) criteria (n = 13 studies)^32,36,38,41,46,61,71,76–79,122,132^. The study by Shu et al*.* used UPDRS III, the H&Y scale and MRI to diagnose their patients^69^.

Most studies reported data on miRNAs which were significantly different between PD patients and controls (*p* < 0.05). However, the studies by Han et al., Schwienbacher et al. and Sethi et al. presented data on miRNAs which did not reach statistical significance^63,92,105^. MicroRNA expressions were reported most commonly from serum and serum-exosomes (n = 35 studies),^10,29–31,33,34,38,43,50,51,54,58,63–66,69,72,75,76,81–84,87,91,98,100,107–110,122,126,128^ plasma and plasma exosomes (n = 22 studies),^32,41,42,44,45,48,49,60,67,73,77,78,80,95,97,104,105,119,120,125,129,132^, CSF and CSF exosomes (n = 10 studies)^35,39,53,62,68,70–72,85,125^, PBMCs (n = 10 studies)^9,36,59,103,106,115–118,121^, whole blood (n = 3 studies),^8,105,123^, saliva (n = 3 studies),^37,46,47^ a peripheral blood leukocytes (PBLs) (n = 3 study)^56,79,124^. Other miRNA sources included brain tissue (n = 16 studies)^4,57,74,86,88–90,92–94,96,99,102,113,114,127^ and sigmoid colon (n = 1 study),^112^, because of the proposed gastrointestinal origin of PD^131^.

Of the studies on brain tissue from PD patients and controls, the commonly studied regions included the SN (n = 6 studies),^86,88,94,96,99,127^ prefrontal cortex (n = 3 studies),^4,57,74^ midbrain (n = 2 studies),^89,113^, amygdala (n = 2 studies)^94,99^, putamen (n = 2 studies),^90,102^, cerebellum (n = 1 study),^99^, temporal lobe neocortex (n = 1 study),^92^, striatum (n = 1 study),^96^, and anterior cingulate gyrus (n = 1 study)^114^. The study by Cho et al. reported miRNA expression data from the SN and the striatum separately^88^. Additionally, Tolosa et al. reported miRNA expression from dopaminergic neurons generated by reprogramming somatic cells and promoting the differentiation on induced pluripotent stem cells (iPSCs)^101^.

The technique most commonly used for miRNA analysis was quantitative reverse transcription polymerase chain reaction (RT-qPCR) (n = 90 studies) studies^7–10,29–34,36–51,53,54,57–66,68–70,72–84,88–91,94–110,113–128,132^ with 40 studies using individual TaqMan miRNA assays^9,29,30,33,34,39,40,42,46,48,53,62,66,68,72,74,75,82,90,91,94–101,104–107,110,121,122,124,125,127,132^ and 29 studies using SYBR™ green assays^8,31,36,41,43,45,47,50,51,54,59–61,64,65,73,79,80,83,88,114–120,123,128^. Other miRNA quantification techniques included next generation sequencing (NGS) (n = 12 studies),^4,33–35,56,57,67,71,85,87,111,112^ microarrays (n = 15 studies),^8,37,45,48,59,90–93,99,119–121,125,129^ TaqMan Low Density Arrays TLDAs (n = 9 study)^30,62,72,86,95,96,101,107,114^.

Variability was also reported in the miRNA normalization strategies used. The two most common normalizers were stable miRNAs, including synthetic miRNAs (n = 30 studies)^29–33,40–42,45,46,48,54,66,68,77,95,97,98,100,101,104,106,108–110,113,117,119,121,123,132^ and U6 snRNA or other small nucleolar RNAs snoRNAs (n = 45 studies)^9,10,37,40,43,44,47–50,53,59–62,64,65,69,70,72–74,76,78–84,88,92,96,99,101–103,106,114–116,118,124,126,127^.

### Consistently altered biofluid-derived microRNAs.

Despite the growing body of literature on miRNAs in PD patients, there remains limited overlap in biofluid miRNAs reported to be differentially expressed between PD patients and controls. Therefore, there is a need to identify and robustly validate a few key miRNAs which are consistently reported to be changed in PD literature, which could serve as potential diagnostic biomarkers for PD. Bearing this in mind, all differentially expressed miRNAs from biofluid studies, and their directions of change were extracted from studies included in this systematic review. Table 2 presents the 23 miRNAs which have been reported to be differentially expressed by ≥ 3 biofluid studies, along with their biofluid source and directions of change. MiR-331-5p was found to be consistently up-regulated in 5 different sources of biofluid. However, miR-24-3p and miR-214-3p were the most reported miRNA in different studies (7 each). Only miR-19b-3p, miR-24-3p, miR-146a-5p, and miR-221-3p were identified to be reported without conflicting directions of change in the same biofluid type (serum) in multiple studies. Therefore, these miRNAs could be considered as strong candidates for future biomarker studies on PD.

**Table 2:**
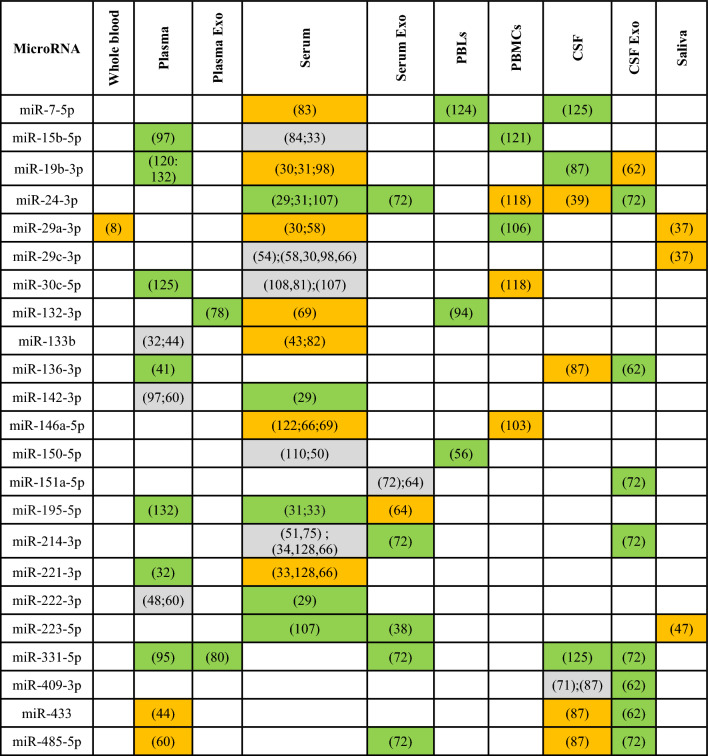
MicroRNAs reported to be significantly changed between PD and controls reported by ≥ 3 biofluid studies.

Green indicates an up-regulation, orange a down-regulation and grey a conflict of direction within the same sample type.

### Pathway analysis of key biofluid-derived microRNAs

**Figure 2:**
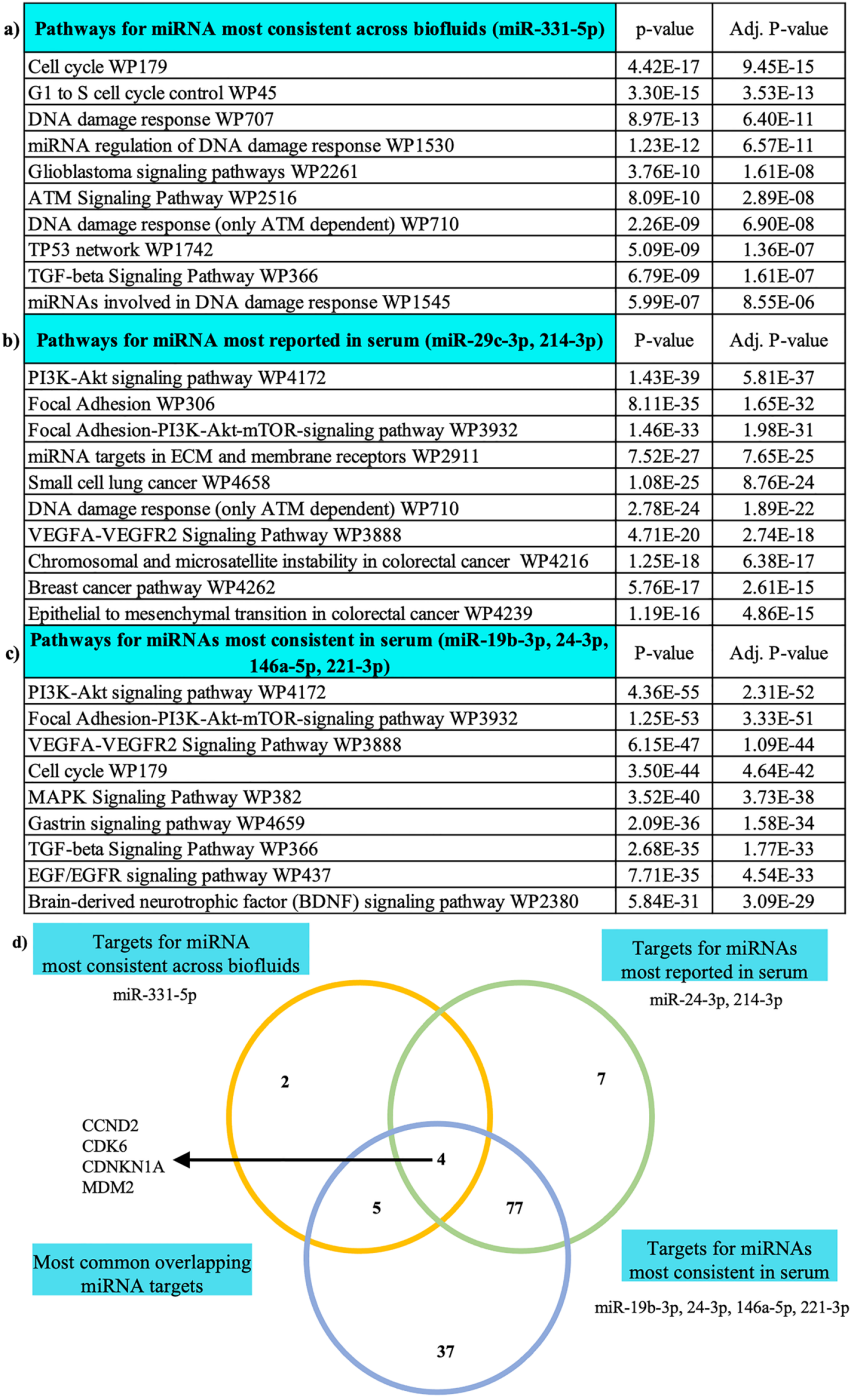
Bioinformatic analysis of key biofluid-derived microRNAs. Top enriched pathways (Fisher exact test *p* < 0.05) associated with (**a**) most consistent miRNA across biofluids, miR-331-5p (**b**) most reported miRNA, miR-24-3p and miR-214-3p and (**c**) miRNA reported without conflicting direction of change, miR-19b-3p, miR-24-3p, miR-146a-5p, and miR-221-3p and (**d**) Venn Diagram showing the target genes regulated by the key biofluid-derived microRNAs. Overlapping target genes: CCND2 (Cyclin D2), CDK6 (Cyclin Dependent Kinase 6), CDNKN1A (cyclin dependent kinase inhibitor 1A) and MDM2 (Mouse Double Minute 2).

We next explored potential biological relevance of the most consistently altered miRNAs: (1) miR-331-5p because of its consistency in multiple biofluids, (2) miR-24-3p and miR-214-3p as the most reported in serum and (3) miR-19b-3p, miR-24-3p, miR-146a-5p, and miR-221-3p because of their consistency in serum. We first identified the validated gene targets of these three groups using DIANA mirPathv3-TarBase (gene union algorithm) and then the most highly enriched pathways associated with the gene targets using Enrichr (Fig. 2a–c). This analysis showed that the functions of the identified miRNAs relate to pathways previously associated with PD pathology, such as PI3K-signalling and cell cycle control e.g.,^133,134^. Next, exploring the target genes associated with the enriched pathways, we found 4 target genes common to all pathways (CCND2, CDK6, CDNKN1A, and MDM2; Fig. 2d), including 3 molecules involved in cyclin-dependent pathways [CCND2 (Cyclin D2), CDK6 (Cyclin Dependent Kinase 6), CDNKN1A (cyclin dependent kinase inhibitor 1A)], which have a strong association with degeneration of dopaminergic neurons in PD. Further investigation showed that MDM2 is targeted by 5 of the 6 miRNAs contributing to the enriched pathways. MDM2 encodes an E3 ubiquitin ligase which enhances Parkin activity and has promise as a potential anti-parkinsonian therapy^135^. Together these analyses suggest that the identified biofluid-derived miRNAs, may be capable of reflecting the PD-related neurodegenerative processes.

### Meta-analysis

Next drawing on available AUC, sensitivity and specificity we performed meta-analyses. A total of 25 biofluid studies provided diagnostic accuracy data on (a) combination of miRNAs or (b) individual miRNAs from PD patients and controls (Table 1)^29–39,41–51,53,54,65^. The included studies originated either from China (n = 14 studies), European countries (n = 6), North America (n = 3), or rest of Asia (n = 2). A total of 7 studies^30,37,41,44,45,48,49^ specified including sporadic PD patients in their cohort and 6 studies^29,37,38,43,47,54^ reported data on treated PD patients. The most common diagnostic criteria used in the included studies was the UKPDSBBC n = 14 studies^30,31,35,41,44,45,47–51,53,54,65^. Additionally, all studies, except Dos Santos et al., used RT-qPCR as their method of miRNA analysis, with the most common normalizer in these studies being stable miRNAs n = 12 studies^29–34,41,42,45,46,48,54^. Of the included studies, 11 were conducted on serum-derived miRNAs^29–31,33,34,38,43,50,51,54,65^, 7 on plasma-derived miRNAs^32,41,42,44,45,48,49^, 3 on CSF-derived miRNAs^35,39,53^, 3 on saliva-derived miRNAs^37,46,47^ and 1 on PBMCs-derived miRNAs^36^.

**Figure 3:**
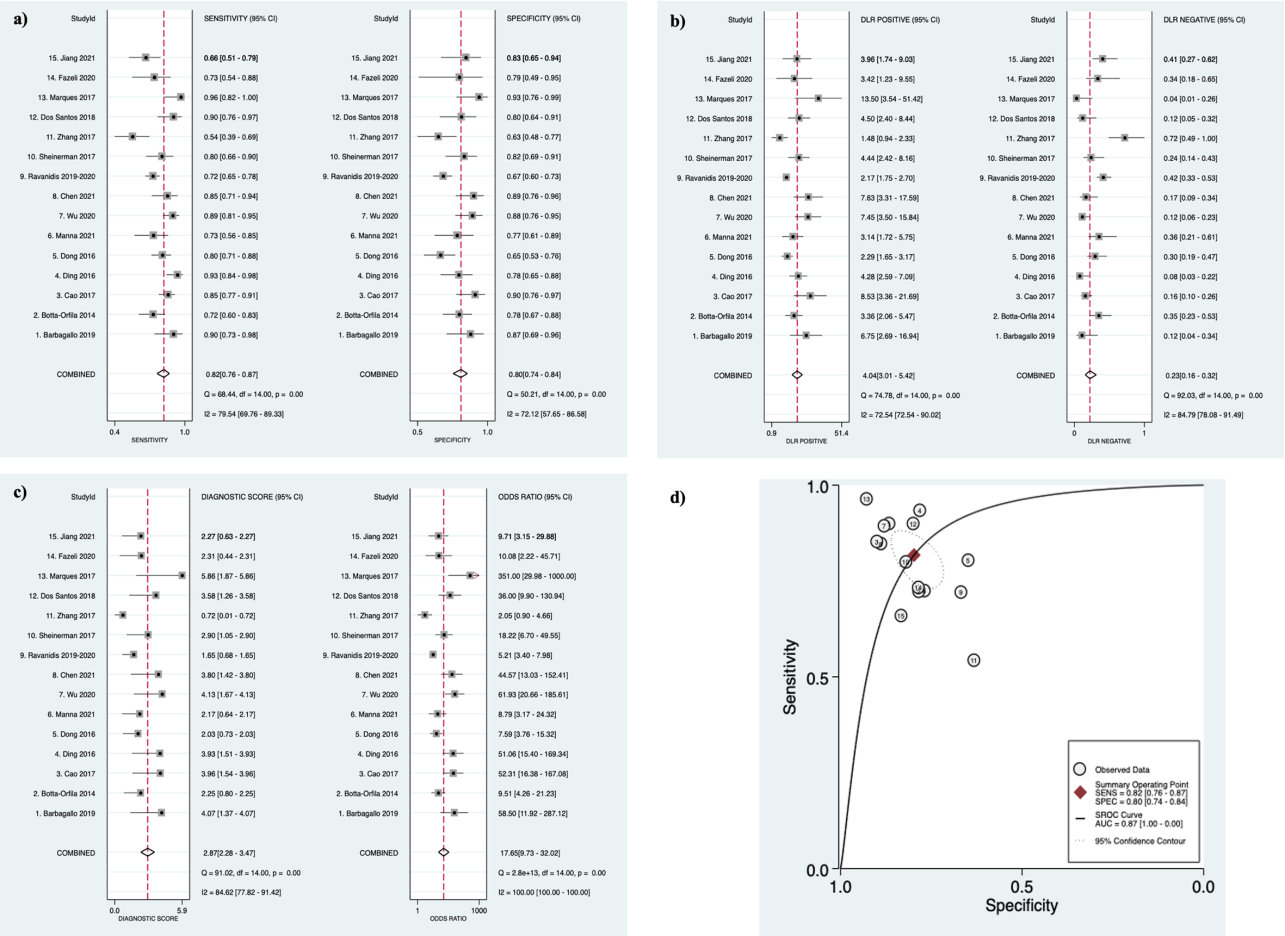
Diagnostic accuracy indices for meta-analysis of the combinations of miRNAs. (**a**) sensitivity and specificity of biofluid-derived microRNAs with corresponding estimates for heterogeneity I2 tests (n = 15 studies). The sensitivity or specificity for each study is represented by the black circles in the grey square and the 95% confidence interval for each study is represented by the associated horizontal lines. (**b**) positive and negative likelihood ratios: PLR/NLR is represented by the black circles in the grey squares and the 95% confidence interval for each study is represented by the associated horizontal lines (**c**) diagnostic score and diagnostic odds ratio: the diagnostic score/DOR are represented by the black circles in the grey squares and the 95% confidence interval for each study is represented by the associated horizontal lines. The pooled estimate is represented by the diamonds with the horizontal edges of the diamonds reflecting the 95% confidence interval. The red dotted line represents the pooled average point estimate. The studies are ordered by biofluid type: Serum^1–7^, Plasma^8–11^, CSF^12,13^, PBMC^14^ and Saliva^15^. (**d**) Summary Receiver Operating Characteristic curve of biofluid-derived microRNAs in PD patients compared to controls (n = 15 studies). The red filled diamond represents the summary estimate of the test accuracy, and the dotted line denoting the 95% confidence region around this estimate. The circles represent each study involved in the meta-analysis: 1^29^, 2^30^, 3^31^, 4^33^, 5^34^, 6^38^, 7^43^, 8^32^, 9^41^, 10^42^, 11^44^, 12^35^, 13^39^, 14^36^, 15^37^.

For the meta-analyses, the 15 studies^29–39,41–44^ providing diagnostic accuracy data on miRNA combinations were pooled and the 17 studies providing data on individual miRNAs were analyzed in a separate meta-analysis^29,31–33,36,37,43,45–51,53,54,65^. Combining the 15 studies providing diagnostic accuracy data on miRNA combinations allowed assessment of 980 PD patients and 802 controls. This analysis demonstrated the following diagnostic accuracy indices: sensitivity 0.82 (95% CI 0.76–0.87), specificity 0.80 (95% CI 0.74–0.84) (Fig. 3a), PLR 4.04 (95% CI 3.01–5.42), NLR 0.23 (95% CI 0.16–0.32) (Fig. 3b), diagnostic score 2.87 (95% CI 2.28–3.47) and DOR 17.65 (95% CI 9.73–32.02) (Fig. 3c). This demonstrates a good overall diagnostic performance. The I*2* test of overall heterogeneity was 88.95% (95% CI 76%-100%) and the Chi-square test was calculated to be 17.182 (*p* = 0.000). Combining sensitivity and specificity for all 15 studies enabled the creation of the SROC curve with an associated strong AUC value of 0.87 (95% CI 0.83–0.89) (Fig. 3d).

The Fagan’s nomogram which includes pre-test and post-test probabilities of developing PD based on the calculated likelihood ratios of these miRNAs and the associated PPV and NPV values is presented in Supplementary Figure S2. According to the constructed Fagan’s nomogram, the calculated pre-test probability of PD was 50%. Using these miRNAs, a positive test, and a PLR of around 4 would elevate the post-test probability of PD to 80%. With a negative result and an NLR of 0.23, the miRNAs would reduce the post-test probability to 19%. Additionally, the probability-modifying plot demonstrated a PPV and NPV of 0.78 (95% CI 0.75–0.82) and 0.80 (95% CI 0.76–0.84), respectively. Therefore, based on the likelihood ratios and the post-test probabilities, these miRNAs have potential to improve the diagnostic efficiency of PD.

### Heterogeneity

**Figure 4:**
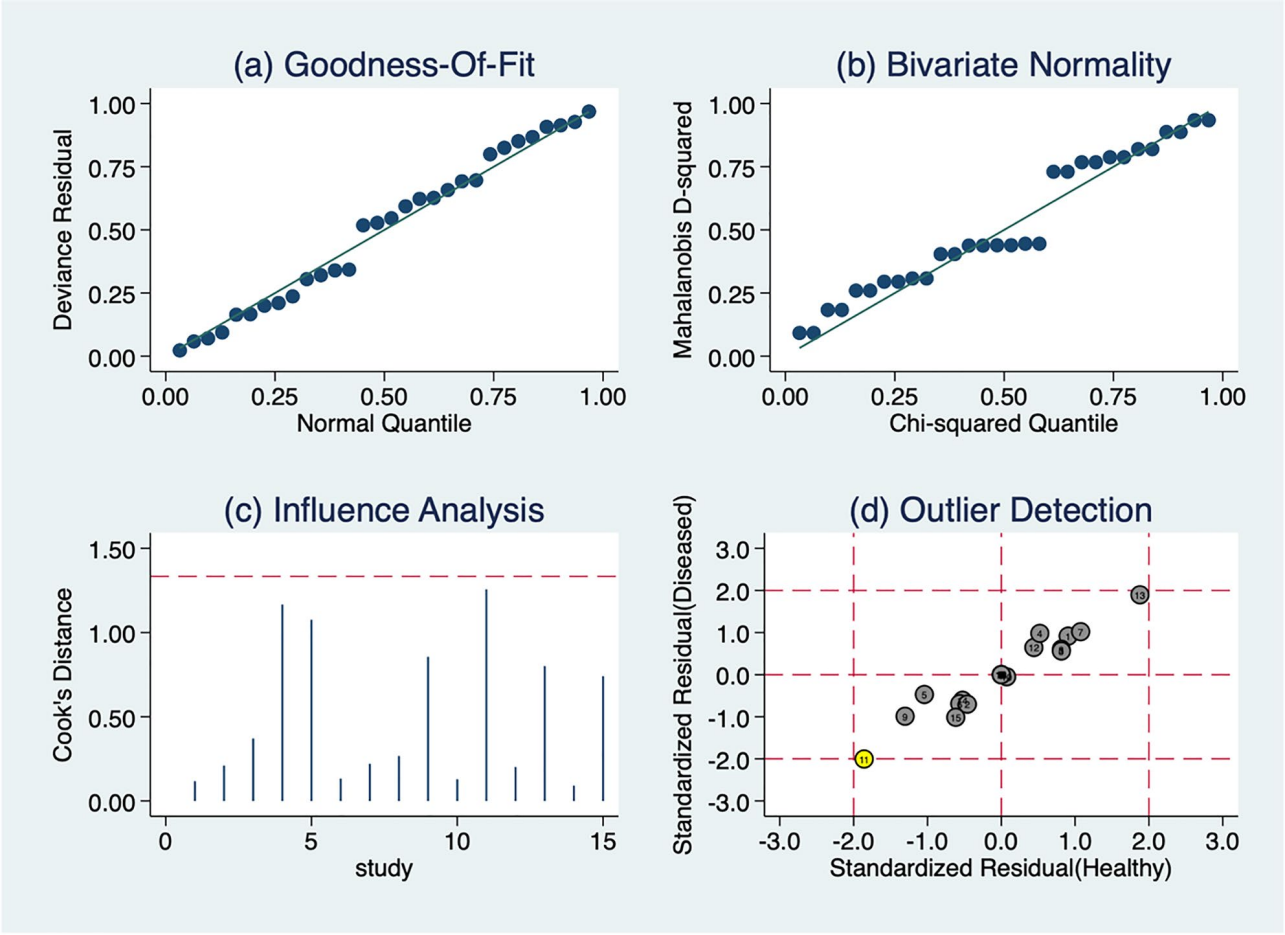
Model diagnostics to assess suitability and robustness of the bivariate model. Graphical representation of (**a**) goodness of fit (quantile plot of residual) (**b**) bivariate normality (Chi-squared probability plot of squared Mahalanobis distances), (**c**) influence analysis (spike plot using Cook’s distance; threshold = 4/n where n is the total number of data points;) and (**d**) outlier detection (scatter plot using standardized predicted random effects); yellow: = study outside the 95% boundaries and may be considered as an outlier. These analyses were conducted on biofluid-derived microRNAs from PD patients and controls (n = 15 studies). The circles represent each study involved in the meta-analysis: 1^29^, 2^30^, 3^31^, 4^33^, 5^34^, 6^38^, 7^43^, 8^32^, 9^41^, 10^42^, 11^44^, 12^35^, 13^39^, 14^36^ and 15^37^.

Substantial heterogeneity (I2 > 50% and Chi-square *p* < 0.10) was identified in all diagnostic accuracy indices in this analysis. Before exploring the sources of heterogeneity, model diagnostics were run to assess the suitability and robustness of the bivariate model and thus assess the reliability of the results of this analysis. As shown in Fig. 4, data from biofluid-derived miRNA studies were assessed using model diagnostics for (a) goodness of fit using a quantile plot to evaluate whether the residuals are normally distributed (b) bivariate normality using a Chi-squared probability plot, for assessment of the bivariate normality assumption. Additionally, (c) a spike plot and Cook’s distance were used for identification of influential studies in regression analysis and (d) a scatter plot was used to identify outliers which could potentially contribute to the substantial heterogeneity found in the meta-analysis.

The Goodness-of-Fit (Fig. 4a) and bivariate normality (Fig. 4b) graphs demonstrated that the model used in this meta-analysis was appropriate (both showed normality). Additionally, no study was deemed to be more influential than others as none reached the threshold (hashed red line in Fig. 4c). Only 1 study^44^ was detected as a potential outlier (yellow circle) (Fig. 4d). However, we did not remove this outlier because it was not deemed more influential than any other study. Therefore, model diagnostics suggested that the bivariate mixed-effects binomial regression model used in the MIDAS platform was appropriate for the data in this analysis and provided valid and robust results.

**Figure 5:**
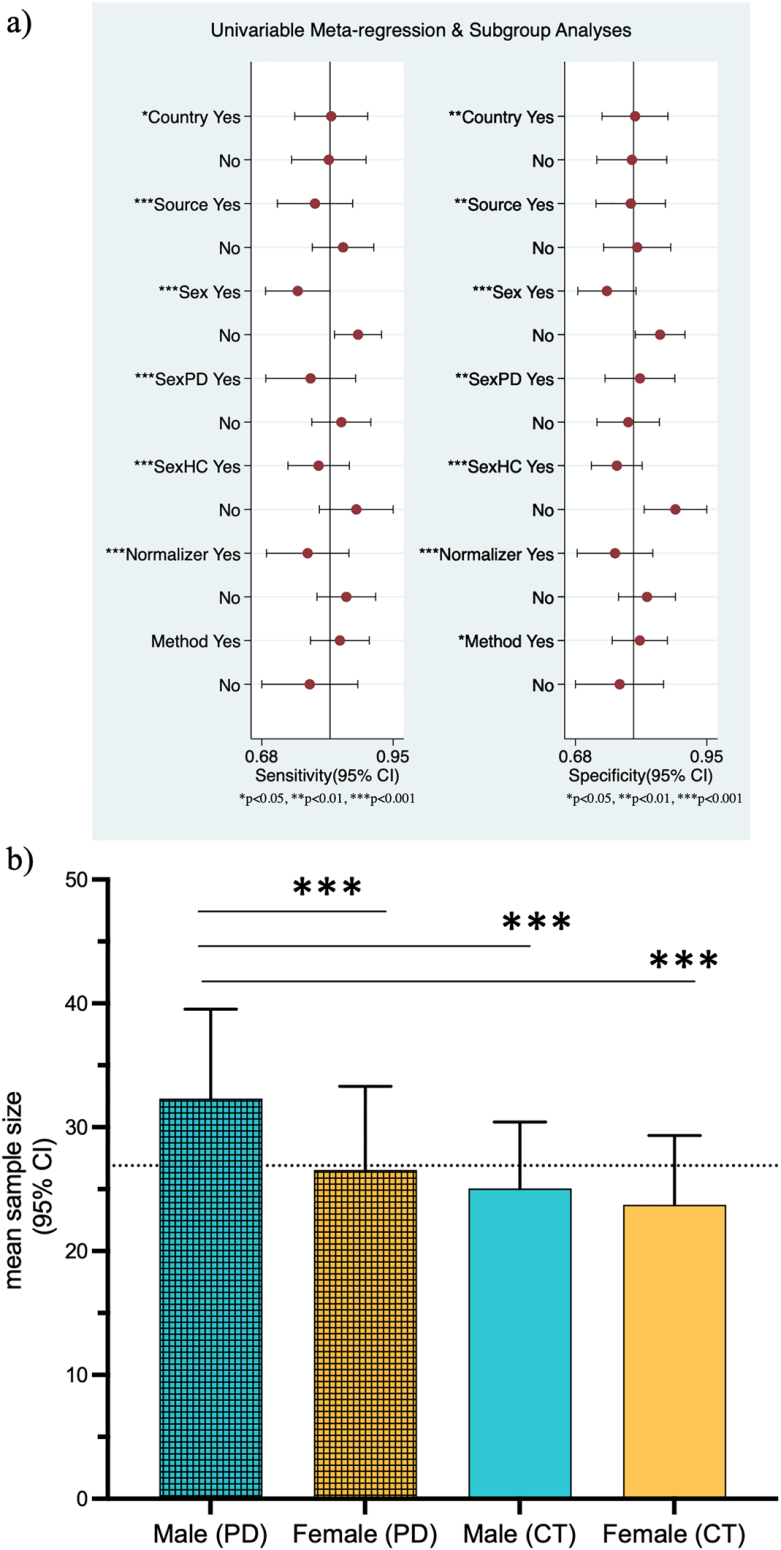
Meta-regression analysis and subgroup analysis (**a**) Meta-regression analysis of study characteristics to identify potential sources of heterogeneity in sensitivity and specificity of biofluid-derived microRNAs from PD patients and controls. Sensitivity and specificity of each study characteristic is represented by the red dot with the corresponding horizontal line showing 95% confidence interval. Vertical line represents the average point estimate. *p < 0.05, **p < 0.01, ***p < 0.001. (**b**) Bar graph showing sex distribution of the groups studied in the systematic review: mean sample size and 95% CI for each group (blue = male, orange = female). The horizontal dotted gray bar represents the mean average sample size for the 4 groups. ***p ≤ 0.001 Dunn’s post hoc tests.

To explore underlying causes of the high heterogeneity in this meta-analysis, firstly assessments were conducted to detect the presence of any threshold effects. The Spearman’s correlation coefficient (r = -0.40, *p* = 0.20) demonstrated that no significant threshold effects contributed to the heterogeneity observed in this meta-analysis. Subgroup analysis and meta-regression were performed to assess how different confounding factors affected the overall diagnostic accuracy and could be potential sources of heterogeneity between the studies included in this meta-analysis. The following parameters were included in the meta-regression: type of biofluid (serum n = 7 vs other biofluids (plasma n = 4, CSF n = 2, saliva n = 1, PBMCs n = 1), sex of participants (# male vs # female) as total in each study (male n = 871 vs female n = 826) and within a group in each study (PD male n = 521 vs PD female n = 400; control male n = 350 vs control female n = 426), country where studies were conducted (China n = 7 vs other countries n = 8), method of miRNA analysis (RT-qPCR TaqMan n = 10 vs others methods n = 5), and how the miRNA expression was normalized (stable miRNA n = 7 vs others methods n = 8 ) (Fig. 5a).

Meta-regression analysis identified that the following covariates may all have contributed to the source of heterogeneity: the sample type used, country where studies were performed, how the miRNA expression was normalized, sample size of each sex of the participants either when looked as a whole or within each group; these all contributed to heterogeneity in both specificity and sensitivity (Fig. 5a). However, the method of analysis for miRNA expression only seemed to have affected the specificity. Interestingly, when the joint model was analyzed, it appears that the main cause of heterogeneity was found in the total sex distribution; overall more men than women participants regardless of group (*I*^2^ = 76 *p* = 0.01), and sex distribution within control group; overall more women than men participants int the control group (*I*^2^ = 63 *p* = 0.06). The sex distribution in the PD group and normalizer used in miRNA expression analysis also showed heterogeneity but these were not significant ((*I*^2^ = 51; *p* = 0.13; *I*^2^ = 24; *p* = 0.27, respectively).

### Sub-group analysis

We performed additional analyses to determine whether the diagnostic utility of miRNA in biofluid differed among certain subgroups. The diagnostic accuracy indices of subgroup analyses are presented in Table 3 (pooled estimates including sensitivity, specificity, AUC, and partial AUC for each subgroup). We divided these studies into four subgroups to study the effects of: (1) origin of study, using data from the studies included in the meta-analysis, (2) method of normalization of miRNA expression, (3) type of biofluid and (4) the influence in using a single miRNA versus a panel of miRNAs as a predictors of PD.

**Table 3:** The summary data and performance estimates of subgroup analyses.

Subgroup	Sensitivity	Specificity	PLR	NLR	DOR	AUC	I^2^	Q
	(95% CI)	(95% CI)	(95% CI)	(95% CI)	(95% CI)	(95% CI)		
Origin of study: Panel of miRNAs								
China (n = 7)	0.82	0.8	4.3	0.17	26	0.91	57	4.635
	(0.75–0.89)	(0.73–0.87)	(2.8–6.7)	(0.11–0.25)	(12–56)	(0.88–0.93)	(3–100)	*p* = 0.049
Rest of the world (n = 8)	0.82	0.79	4.5	0.23	19	0.87	63	5.343
	(0.74–0.84)	(0.72–0.87)	(2.8–7.1)	(0.11–0.49)	(6–62)	(0.83–0.89)	(16–100)	*p* = 0.035
Normalization of RT-qPCR data: Panel of miRNAs								
Stable miRNAs (n = 7)	0.85	0.82	4.7	0.18	25	0.86	90	19.33
	(0.73–0.92)	(0.77–0.85)	(3.5–6.3)	(0.1–0.35)	(10–63)	(0.83–0.89)	(79–99)	*p* = 0.000
Other normalization method n = (8)	0.81	0.79	3.8	0.24	16	0.86	67	5.3
	(0.69–0.9)	(0.69–0.86)	(2.3–6.3)	(0.13–0.44)	(5–48)	(0.15–1)	(41–93)	*p* = 0.038
Type of Biofluid: Panel of miRNAs								
Serum (n = 8)	0.82	0.82	4..5	0.21	21	0.89	80	9.979
	(0.71–0.88)	(0.75–0.87)	(3.2–6.4)	(0.14–0.33)	(11–42)	(0.85–0.91)	(57–10)	*p* = 0.003
Other biofluid (n = 7)	0.75	0.82	4.2	0.3	14	0.86	62	5.225
	(0.71–0.8)	(0.78–0.85)	(3.5–5)	(0.25–0.36)	(11–18)	(0.83–0.88)	(14–100)	*p* = 0.037
Combination versus single miRNA								
Combination miRNAs (n = 15 studies)	0.82	0.80	4.04	0.23	17.9	0.87	88.95	17.182
	(0.76–0.87)	(0.74–0.84)	(3–5.4)	(0.16–0.32)	(9–32)	(0.86–0.91)	(76–100)	*p* = 0.000
Single miRNA (n = 17 studies)	0.77	0.8	3.9	0.29	13.46	0.85	93	28.341
	(0.73–0.8)	(0.77–0.84)	(3.25–4.18)	(0.25–0.3)	(11–18)	(0.82–0.88)	(87–99)	*p* = 0.000

We found that studies from China (n = 7) demonstrate slightly higher AUC values than the rest of the world (n = 8) (0.91 vs 0.87) and achieved narrower confidence intervals for all diagnostic accuracy indices. This may underpin the smaller heterogeneity observed in this subgroup. However, as the confidence intervals overlap. this suggests similar overall diagnostic accuracies have been achieved.

The methods of miRNA data normalization used in the included studies were also another significant source of heterogeneity in this analysis. Subgroup analysis of the 7 studies which reported using stable miRNAs as their normalizer demonstrated comparable diagnostic indices to the subgroup analysis of the rest of the normalization method (n = 8) (0.86 vs 0.86). Additionally, the 8 serum studies subgroup also demonstrated diagnostic accuracy indices which were like the overall analysis. This suggests that miRNAs derived from serum might have potential to serve as diagnostic biomarkers of PD. While serum studies appeared to have higher sensitivity (82% vs 75%) than other biofluids, further diagnostic accuracy studies with larger samples sizes are required in order to clarify any significant differences between miRNAs derived from a particular biofluid. Interestingly, multiple miRNAs (panel of miRNAs: 15 studies) appeared to be a better predictor than single miRNA (n = 17 studies), with higher AUC (0.87 vs 0.85), better sensitivity (82% vs 77%), but equal specificity (80% vs 80%) and higher DOR (18 vs 13), an important measure of effectiveness at detecting PD in biofluid.

### Publication bias

Publication bias is generally analyzed using the funnel plot which graphs effect size against study precision with asymmetry of the plot indicating a presence of publication bias. However, due to the subjectivity in the interpretation of funnel plots, methods such as the Deeks’ funnel plot asymmetry test (Supplementary Figure S3) were developed, which conducts a regression of the diagnostic log odds ratio (X-axis) against the inverse square root of effective sample sizes (Y-axis)^136^. The Deeks’ regression line coefficient had a *p* value of 0.054. The Egger’s regression test showed a symmetrical effect size (intercept = 0.71, t = 0.73, *p* = 0.481) therefore both tests suggest there is no significant evidence to support publication bias.

### Selection bias: sex differences in PD

Clinical information indicates that there are clear sex differences in many aspects of PD^137^. Given that we have found an uneven sex distribution in the sample sizes across all studies, and that it contributed to the significant between-study heterogeneities in our meta-analysis, we performed a Friedman test to compare the sample sizes in the four groups followed by Dunn's post test to further investigate the impact caused by this. For this analysis we have used the entire biofluid data in the systematic review because it allowed us to increase the number of studies investigated (n = 89) including 5104 individuals diagnosed with PD (n = 2874 male and female n = 2230) and 4474 controls (male n = 2363; female n = 2112).

We found an overall significant difference between the mean sample size of the 4 groups studied (*p* < 0.0001) (Fig. 5b). The post hoc analysis revealed that there was significant difference between the following pairwise comparison: # male (PD) vs. # female (PD): adj. *p* < 0.0001; # male (PD) vs. # male (HC): adj. *p* = 0.0007; # male (PD) vs. # female (HC): adj. *p* < 0.0001, but not between # male (HC) vs. # female (HC) or # female (PD) vs. # female (HC). Thus, multiple studies failed to achieve a balanced sex representation within their participant groups. In particular, this could underpin a selection bias^138^ in the PD group where overall more males were enrolled compared to females, a difference not as pronounced in the control groups. Overall, this raises concerns about the representativeness and comparability of the control group used in some of these studies.

## Discussion

Early diagnosis of PD, before the onset of significant neurodegeneration, is crucial to developing and testing novel disease-modifying therapies. MiRNAs are ideally suited as biomarkers of neurodegeneration as they are dynamically altered with neuropathology and released from brain cells to enter the blood stream, where they are remarkably stable. The ever-evolving nature of the field of miRNAs in PD mandates that data on newly identified or validated miRNAs must be periodically updated to help strengthen the evidence of the diagnostic potential of miRNAs in diseases such as PD. Moreover, knowing the diagnostic value of miRNAs will be imperative to future clinical decision-making if miRNAs are to be implemented as diagnostic tests for PD. Therefore, this meta-analysis pooled diagnostic accuracy data from 15 studies to demonstrate that the combination of all biofluid miRNAs can discriminate between PD patients and controls with 82% sensitivity, 80% specificity and an AUC of 0.87, suggesting that miRNAs have the potential to serve as useful diagnostic biomarkers for PD.

Although the field of miRNAs in PD diagnosis has expanded substantially in the past few years, there remains a distinct lack of diagnostic accuracy data on the same miRNAs (individual and combinations of miRNAs) from multiple different studies on PD patients and controls. Additionally, due to the lack of accepted standards in the field of miRNAs as biomarkers of diseases, there is still some debate on the value of using individual miRNAs compared to combinations of miRNAs in research studies. In this meta-analysis, single miRNA assays generally had lower AUC and sensitivity values when compared to miRNA combinations, suggesting that multi-miRNA panels could have better diagnostic usefulness in PD. Interestingly, in other neurodegenerative diseases such as multiple sclerosis, meta-analyses have reported higher diagnostic accuracies in individual rather than combinations of miRNAs^14^. This suggests that miRNAs might show disease-specific alterations and should be understood in the context of each neurodegenerative disease individually. However, we think that it is not surprising that multi-miRNA panels performed better because molecular biomarkers do not exist in isolation from each other. Every biological factor is part of a complex matrix of interconnected biological events and therefore each factor’s relationships will affect their predictive powers. If two or more miRNAs are linked to the disease process in different ways, then when placed in the same predictive model, they will be additive in the model and allow more accuracy.

To date, data which allow for the calculation of the 2 × 2 contingency tables have only been reported for miR-24-3p, miR-19b-3p, miR-153-3p and miR-195-5p by two separate studies each, which does not provide sufficient data for pooling into individual meta-analyses^30,31,33,47,62^. Further data on individual miRNAs would also allow for stratification based on direction of change and could perhaps enable clarification on whether individual miRNAs with the same direction of change provide greater diagnostic accuracies compared to miRNA combinations. This demonstrates the need for future studies to validate, report and meta-analyze diagnostic accuracy data on individual miRNAs or combinations of miRNAs which are already known to be able to discriminate between PD patients and controls.

### miR-331-5p and miR-19b-3p, miR-146a, miR-24-3p, miR-221-3p reported by multiple biofluid studies without conflicting directions of change.

Although there is limited diagnostic accuracy data on overlapping miRNAs, the comprehensive pooling of all available studies on miRNAs from PD patients and controls into the systematic review identified miR-331-5p alongside miR-19b-3p, miR-24-3p, miR-146a and miR-221 to be reported by multiple biofluid studies without any conflicting directions of change. These miRNAs, therefore, could be considered as a point of focus for future miRNA biomarker studies on PD patients.

The gene targets of miR-331-5p, identified using bioinformatic analyses, were associated with pathways important for PD pathology including the ataxia-telangiectasia (ATM) signaling pathway. Specifically, ATM kinase has been hypothesized to regulate the function of the PD-related genes PINK1 and Parkin, which are important for mitophagy^139,140^. Additionally, the PINK1 gene also regulates cell cycle progression, another pathway which was found to be highly associated with miR-331-5p^141^. ATM plays a role in signal transduction control and regulates DNA damage response signaling pathway. Although DNA damage response is not traditionally a point of focus in PD pathology, dysfunctional DNA repair and accumulation of DNA damage is considered integral to cellular aging, and aging is the most important risk factor for PD^142,143^. Interestingly, ATM is a member of the phosphatidylinositol 3-kinase (PI3K)-signaling family, and PI3K was a shared biological function identified in our analyses of the miRNA reported most commonly and those in multiple biofluid studies without conflicting directions of change. This family is tightly linked to apoptosis, the primary mechanism behind the neuronal cell death associated with PD, as demonstrated by post-mortem studies on PD brains which show apoptosis-like changes in dopaminergic neurons^144^ and miR-19b and miR-221-3p have been specifically implicated in apoptosis^145,146^. Furthermore, both miR-146a-5p and miR-24-3p effect mitophagy the process of removing damaged mitochondria which is impaired in PD. miR-146a-5p has been shown to enhance mitochondrial dysfunction via reducing Parkin levels and miR-24-3p sponges have been shown to enhance mitophagy and therefore promote elimination of damaged mitochondria^147^. Additionally, miR-214-3p has been shown to inhibit α-synuclein expression, which is the major component of Lewy bodies one of the pathogenic hallmarks of PD^148^. Alongside this, several studies included in this systematic review have also reported other miRNAs which are associated with pathways important in PD pathology. For example, downregulation of miR-133b has been demonstrated in PD brains. This miRNA is postulated to play a role in PD by regulating the transcription factor Pitx3, involved in dopamine neuron development^79,113^. This miRNA has also been shown to be downregulated in the plasma and serum of PD patients^43,44,82^ Therefore, the clustering of several related pathways highlights the potential of peripheral biofluid miRNAs to both identify those at risk of developing PD and to further our understanding of CNS pathologies.

### Diagnostic accuracy of miRNAs in individual biofluids

The diagnostic accuracy of miRNA in individual biofluids was also assessed in the current meta-analysis. Subgroup analysis on serum-derived miRNAs demonstrated a sensitivity and specificity of 82% with an AUC of 0.89. Due to insufficient studies on plasma and CSF-derived miRNAs, no conclusions could be drawn on miRNAs isolated from these biofluids. However, plasma and serum-based tests are already widely used in current clinical practice, making them ideally suited to serve as sources of miRNAs in diagnostic studies^149^. MiRNAs have shown stability in plasma and serum by resisting degradation by endogenous RNases^150^. They have also been shown to maintain this stability through multiple freeze–thaw cycles^151,152^. However, differences in these biofluids have been reported by Wang et al., who found higher serum-miRNA concentrations compared to plasma, possibly because of the effect of coagulation on the profiles of miRNA available in blood^153^. Contrastingly, others have found elevated plasma miRNAs compared to serum^150,154^. These inconsistencies support the fact that sample types and analysis methods may not be used interchangeably. However, identifying serum- or plasma-specific miRNA signatures would enable the parallel assessment of both these biofluids which could be collected simultaneously in patients and help strengthen the accuracy and reliability of the diagnosis of diseases such as PD.

Per this systematic review, most studies were conducted on plasma or serum miRNAs from PD patients. Interestingly, the miRNAs from these biofluids have been shown to correlate with clinical and demographical parameters in PD. For example, Botta-Orfila et al. found that downregulated levels of serum miR-29a and miR-29c were significantly associated with the male sex in idiopathic PD patients^30^. Additionally, downregulated levels of the miR-29 family (miR-29a/b/c) have also been reported to correlate with cognitive decline in PD patients with MCI and dementia^63^. Additionally, Ma et al. demonstrated that downregulations of miR-221 in PD serum were positively correlated with UPDRS-III and UPDRS-IV scores, suggesting a potential for the use of miR-221 as a marker of PD severity^66^. MicroRNA levels have also been shown to correlate with antiparkinsonian treatments. For example, Caggiu et al. found decreased miR-155-5p levels in PD patients taking > 458 mg/day of levodopa^103^. Deep brain stimulation has also been reported to alter miRNA expression^56^. Although the exact nature of the interactions of PD therapy with miRNAs remains incompletely understood, this suggests that miRNAs may have the potential to serve as biomarkers to monitor the effectiveness of current and future PD treatment.

Besides plasma and serum, CSF and its unique proximity to the brain makes it a promising biofluid source for miRNAs capable to reflecting neurodegenerative changes in the brain. Interestingly, PBMCs have been theorized to have the greatest potential to reflect brain pathology as these cells share a significant amount of their transcriptome with other cell types, including cells in the CNS^155^. However, this systematic review identified several CSF-based studies which demonstrated an interesting trend of mirroring changes in the CNS. For example, upregulated levels of CSF miR-205-5p was reported by Marques et al.^39^. This miRNA was previously reported to be downregulated in both the SN and the striatum^88^. This trend was also reflected in the upregulated levels of miR-7-5p and miR-218-5p in the CSF and downregulations in the SN and prefrontal cortex^74,125,156^. However, the relative invasiveness of lumbar punctures limits CSF as a routine source of diagnostic biomarkers.

Interestingly, serum and plasma-based studies also demonstrated this trend. For example, miR-124-3p, miR-132-3p and miR-433-3p were found to be upregulated in plasma but downregulated in the prefrontal cortex^4,40,78^. Additionally, downregulations of miR-15b-5p, miR-29a-3p and miR-221-3p were reported in plasma with upregulations reported in the putamen, anterior cingulate gyrus and prefrontal cortex^4,30,33,58,66,90,114^. This suggests that although CSF is advantageously placed in proximity to the brain and believed to be more suited to identifying CNS changes, plasma and serum miRNAs may also be capable of reflecting brain pathology.

Recent studies have also been investigating biofluid-derived extracellular vesicles such as exosomes as biomarkers of PD. For example, Yang et al. found upregulation of miR-135a in serum exosomes, whereas Yao et al. reported elevations in miR-331-5p levels and decreased miR-505 levels in plasma exosomes from PD patients^76,80^. Dos Santos et al. found significant increases in miR-151a-3p and let-7f.-5p and downregulation of miR-27a-3p, miR-125a-5p and miR-423-5p in CSF-derived exosomes^33^. On the other hand, Gui et al. identified and validated the overexpression of miR-10-5p, miR-153, miR-409-3p, and let-7 g-3p, and the downregulation of miR-1 and miR-19b-3p in CSF PD exosomes^62^. Interestingly, the opposite direction of change for miR-10a-5p, miR-19b-3p and miR-409-3p was detected in the CSF by Burgos et al.^87^. This discrepancy could potentially be explained by the differences in the sample population Burgos et al. used CSF sourced post-mortem, whereas Gui et al. had a live patient cohort. Furthermore, the differences in methodology (NGS vs TLDAs) in the two studies might also have influenced the outcomes.

Although this systematic review and meta-analysis provided important insight into the diagnostic value of miRNAs in PD, there were several limitations. Firstly, there was inconsistent reporting of the clinical diagnostic criteria used to diagnose PD patients, as well as incomplete information on medications being received by patients. Furthermore, the disease durations of the included patients showed great variability. However, if miRNAs were to serve as diagnostic biomarkers of PD in the future, this would be reflective of the typical clinical setting where patients might require assessment at varying disease stages, who might be diagnosed using a variety of clinical criteria and be on several different medications. It should be noted, however, that identification of miRNAs corresponding to different disease stages will be advantageous for patient stratification in future PD drug trials.

Secondly, the number of studies available for inclusion were also limited. Additionally, studies also presented with varying sample sizes. Thirdly, the methods of RNA and exosome isolation, and downstream miRNA detection, quantification and normalization methods also varied between studies, with study origin and normalizer being identified as significant sources of heterogeneity in this meta-analysis. Fourthly, there were limited data available on individual miRNAs and their directions of change. Finally, there is little known about sex and gender or ethnicity effects on miRNA biomarkers in PD. Indeed, we identified a significant selection bias, where sex ratios were unevenly distributed between the PD and HC groups, which may confound conclusions drawn. Matching numbers of male and female participants is particularly important as females have a longer life-expectancy and age is a key risk factor for PD. Further, sex differences exist in the prevalence of conditions such as depression, anxiety, and sleep disturbance, all of which are associated with increased risk of developing PD.

## Conclusion

In conclusion, the early diagnosis of PD is hindered by the lack of an accurate diagnostic test. Early or pre-symptomatic diagnosis will be a prerequisite for developing novel disease-modifying therapies capable of preventing disease progression and maintaining good quality of life in patients. This meta-analysis suggests that biofluid-derived miRNAs are promising candidate biomarkers for PD diagnosis which demonstrate good diagnostic accuracy in discriminating between PD patients and controls. The utility of miRNAs as biomarkers lies in their stability and the ease with which they can be accessed from various biofluids. Additionally, although there is limited overlap between the miRNAs reported in PD literature, this systematic review identified miR-331-5p to be reported by multiple biofluid studies, with consistent directions of change, representing a focus point for future biomarker studies. Moreover, the gene targets of these miRNAs all associated with DNA damage and repair pathways, dysfunctions of which have been associated with PD, suggesting the potential of peripheral miRNAs to be able to provide insight into PD pathology.

Therefore, larger studies would help increase the accuracy of the diagnostic results presented in this meta-analysis and provide clarification on whether individual or combinations of miRNAs demonstrate better diagnostic accuracies. Such signatures may be strengthened by general markers of neurodegeneration such as neurofilament light. Additionally, further studies including cohorts which stratify patients based on factors such as sex, ethnicity, disease duration and severity, and symptom severity, would also allow for identification of more specific miRNA signatures. Furthermore, longitudinal studies analyzing miRNA expression changes would enable the identification of miRNA signatures which are able to predict the development of symptoms such as dementia or mood disorders. Independent studies validating already identified miRNAs would potentially allow for a consensus on the most reliable method of miRNA quantification and analysis, help overcome any inconsistencies in direction of change and narrow focus on a key set of miRNAs with the potential to serve as diagnostic biomarkers of PD. Determining disease stage, sex and ethnicity and demographic relevant indicators is the next challenge in diagnostic fluid biomarker research.

### Supplementary Information

Supplementary Information.

## Data Availability

All data generated or analyzed during this study are included in this published article or its supplementary information files.

## References

[1] Nussbaum RL, Ellis CE (2003). Alzheimer’s disease and Parkinson’s disease. N. Engl. J. Med..

[2] Dauer W, Przedborski S (2003). Parkinson’s disease. Neuron.

[3] Singh A, Sen D (2017). MicroRNAs in Parkinson’s disease. Exp. Brain Res..

[4] Hoss AG, Labadorf A, Beach TG, Latourelle JC, Myers RH (2016). MicroRNA profiles in Parkinson’s disease prefrontal cortex. Front. Aging Neurosci..

[5] da Silva F (2016). MicroRNAs involved in Parkinson’s disease: A systematic review. Mol. Med. Rep..

[6] Cogswell JP (2008). Identification of miRNA changes in Alzheimer’s disease brain and CSF yields putative biomarkers and insights into disease pathways. J. Alzheimers Dis..

[7] Rao P, Benito E, Fischer A (2013). MicroRNAs as biomarkers for CNS disease. Front. Mol. Neurosci..

[8] Margis R, Margis R, Rieder CRM (2011). Identification of blood microRNAs associated to Parkinson’s disease. J. Biotechnol..

[9] Fu Y, Zhen J, Lu Z (2017). Synergetic neuroprotective effect of docosahexaenoic acid and aspirin in SH-Y5Y by inhibiting miR-21 and activating RXRα and PPARα. DNA Cell Biol..

[10] Jin L (2018). Elevated microRNA-520d-5p in the serum of patients with Parkinson’s disease, possibly through regulation of cereloplasmin expression. Neurosci. Lett..

[11] De Smaele E, Ferretti E, Gulino A (2010). MicroRNAs as biomarkers for CNS cancer and other disorders. Brain. Res..

[12] Schulz J (2019). Meta-analyses identify differentially expressed microRNAs in Parkinson’s disease. Ann. Neurol..

[13] Hu Y-B (2016). Diagnostic Value of microRNA for Alzheimer’s disease: A systematic review and meta-analysis. Front. Aging Neurosci..

[14] Zhou Z, Xiong H, Xie F, Wu Z, Feng Y (2020). A meta-analytic review of the value of miRNA for multiple sclerosis diagnosis. Front. Neurol..

[15] Zhang W (2022). Circulating microRNAs as potential biomarkers for the diagnosis of Parkinson’s disease: A meta-analysis. Neurologia.

[16] Moher D, Liberati A, Tetzlaff J, Altman DG (2009). Preferred reporting items for systematic reviews and meta-analyses: The PRISMA statement. PLoS Med..

[17] Kozomara A, Birgaoanu M, Griffiths-Jones S (2019). MiRBase: from microRNA sequences to function. Nucleic Acids Res..

[18] Whiting PF (2011). QUADAS-2: A Revised tool for the quality assessment of diagnostic accuracy studies. Ann. Intern. Med..

[19] Vlachos IS (2015). DIANA-TarBase v7.0: indexing more than half a million experimentally supported miRNA:mRNA interactions. Nucleic Acids Res..

[20] Huang H-Y (2022). MiRTarBase update 2022: An informative resource for experimentally validated miRNA–target interactions. Nucleic Acids Res..

[21] Chen EY (2013). Enrichr: interactive and collaborative HTML5 gene list enrichment analysis tool. BMC Bioinform..

[22] Pico AR (2008). WikiPathways: Pathway editing for the people. PLoS Biol..

[23] StataCorp. Stata Statistical Software: Release 16. Preprint at (2019).

[24] Reitsma JB (2005). Bivariate analysis of sensitivity and specificity produces informative summary measures in diagnostic reviews. J. Clin. Epidemiol..

[25] Šimundić A-M (2009). Measures of diagnostic accuracy: Basic definitions. EJIFCC..

[26] Whiting PF (2008). Graphical presentation of diagnostic information. BMC Med. Res. Methodol..

[27] Caraguel CGB, Vanderstichel R (2013). The two-step Fagan’s nomogram: ad hoc interpretation of a diagnostic test result without calculation. Evidence Based Med..

[28] Akobeng AK (2007). Understanding diagnostic tests 2: likelihood ratios, pre- and post-test probabilities and their use in clinical practice. Acta Paediatr..

[29] Barbagallo C (2020). Specific signatures of serum miRNAs as potential biomarkers to discriminate clinically similar neurodegenerative and vascular-related diseases. Cell Mol. Neurobiol..

[30] Botta-Orfila T (2014). Identification of blood serum micro-RNAs associated with idiopathic and *LRRK2* Parkinson’s disease. J. Neurosci. Res..

[31] Cao X-Y (2017). MicroRNA biomarkers of Parkinson’s disease in serum exosome-like microvesicles. Neurosci. Lett..

[32] Chen Q (2021). Elevated plasma miR-133b and miR-221-3p as biomarkers for early Parkinson's disease. Sci. Res..

[33] Ding H (2016). Identification of a panel of five serum miRNAs as a biomarker for Parkinson’s disease. Parkinsonism Relat. Disord..

[34] Dong H (2016). A panel of four decreased serum microRNAs as a novel biomarker for early Parkinson’s disease. Biomarkers.

[35] Dos Santos MCT (2018). miRNA-based signatures in cerebrospinal fluid as potential diagnostic tools for early stage Parkinson’s disease. Oncotarget.

[36] Fazeli S (2020). A compound downregulation of *SRRM2* and miR-27a-3p with upregulation of miR-27b-3p in PBMCs of Parkinson's patients is associated with the early stage of disease. PLoS ONE.

[37] Jiang Y (2021). Profiling of differentially expressed microRNAs in saliva of Parkinson's Disease patients. Front. Neurol..

[38] Manna I (2021). Exosomal miRNA as peripheral biomarkers in Parkinson’s disease and progressive supranuclear palsy: A pilot study. Parkinsonism Relat. Disord..

[39] Marques TM (2017). MicroRNAs in cerebrospinal fluid as potential biomarkers for Parkinson’s Disease and multiple system atrophy. Mol. Neurobiol..

[40] Ravanidis S (2020). Circulating brain-enriched microRNAs for detection and discrimination of idiopathic and genetic Parkinson’s disease. Movement Disord..

[41] Ravanidis S (2020). Validation of differentially expressed brain-enriched microRNAs in the plasma of PD patients. Ann. Clin. Transl. Neurol..

[42] Sheinerman KS (2017). Circulating brain-enriched microRNAs as novel biomarkers for detection and differentiation of neurodegenerative diseases. Alzheimers Res. Ther..

[43] Wu L (2020). Serum miR-9a and miR-133b, diagnostic markers for Parkinson's sisease, are up-regulated after Levodopa treatment. Acta Medica Mediterranea..

[44] Zhang X (2017). Reduced circulating levels of miR-433 and miR-133b are potential biomarkers for Parkinson’s disease. Front. Cell Neurosci..

[45] Chen Y (2017). MicroRNA-4639 is a regulator of DJ-1 expression and a potential early diagnostic marker for Parkinson’s disease. Front. Aging Neurosci..

[46] Chen Y (2020). Increased salivary microRNAs that regulate DJ-1 gene expression as potential markers for Parkinson's disease. Front. Aging Neurosci..

[47] Cressatti M (2020). Salivary microR-153 and microR-223 levels as potential diagnostic biomarkers of idiopathic Parkinson’s disease. Movement Disord..

[48] Khoo SK (2012). Plasma-based circulating microRNA biomarkers for Parkinson’s disease. J. Parkinsons Dis..

[49] Li N (2017). Plasma levels of miR-137 and miR-124 are associated with Parkinson’s disease but not with Parkinson’s disease with depression. Neurol. Sci..

[50] Li H (2020). MicroRNA-150 serves as a diagnostic biomarker and is involved in the inflammatory pathogenesis of Parkinson’s disease. Mol. Genet. Genomic Med..

[51] Li L (2021). Serum miR-214 serves as a biomarker for prodromal Parkinson’s disease. Front. Aging Neurosci..

[52] Lin X (2022). Diagnostic performance of miR-485-3p in patients with Parkinson’s Disease and its relationship with neuroinflammation. Neuromol. Med..

[53] Mo M (2017). MicroRNA expressing profiles in A53T mutant alpha-synuclein transgenic mice and Parkinsonian. Oncotarget.

[54] Ozdilek B, Demircan B (2020). Serum microRNA expression levels in Turkish patients with Parkinson’s disease. Int. J. Neurosci..

[55] Chatterjee P, Roy D (2017). Comparative analysis of RNA-Seq data from brain and blood samples of Parkinson’s disease. Biochem. Biophys. Res. Commun..

[56] Soreq L (2013). Small RNA sequencing-microarray analyses in Parkinson leukocytes reveal deep brain stimulation-induced splicing changes that classify brain region transcriptomes. Front. Mol. Neurosci..

[57] Wake C (2016). Novel microRNA discovery using small RNA sequencing in post-mortem human brain. BMC Genomics.

[58] Bai X (2017). Downregulation of blood serum microRNA 29 family in patients with Parkinson’s disease. Sci. Rep..

[59] Chen Y (2016). Aberration of miRNAs Expression in Leukocytes from Sporadic Amyotrophic Lateral Sclerosis. Front. Mol. Neurosci..

[60] Chen L (2018). Identification of aberrant circulating miRNAs in Parkinson’s disease plasma samples. Brain Behav..

[61] Gong, X., Huang, M., & Chen, L. eNeuro. 2022 Jan 25;91. pi: ENEURO.0393-21.2021.

[62] Gui Y, Liu H, Zhang L, Lv W, Hu X (2015). Altered microRNA profiles in cerebrospinal fluid exosome in Parkinson disease and Alzheimer disease. Oncotarget.

[63] Han L (2020). Association of the serum microRNA-29 family with cognitive impairment in Parkinson’s disease. Aging.

[64] He S (2021). Several miRNAs derived from serum extracellular vesicles are potential biomarkers for early diagnosis and progression of Parkinson's disease. Transl. Neurodegener..

[65] Lin X (2022). Diagnostic performance of miR-485-3p in patients with Parkinson's Disease and its relationship with neuroinflammation. Neuromol. Med..

[66] Ma W (2016). Serum miR-221 serves as a biomarker for Parkinson’s disease. Cell Biochem. Funct..

[67] Nie C (2020). Differential expression of plasma exo-miRNA in neurodegenerative diseases by next-generation sequencing. Front. Neurosci..

[68] Qin L (2019). Preliminary study of hsa-miR-626 change in the cerebrospinal fluid of Parkinson’s disease patients. J. Clin. Neurosci..

[69] Shu Y, Qian J, Wang C (2020). Aberrant expression of microRNA-132-3p and microRNA-146a-5p in Parkinson’s disease patients. Open Life Sci..

[70] Su Y (2019). MicroRNA-26a/death-associated protein kinase 1 signaling induces synucleinopathy and dopaminergic neuron degeneration in Parkinson’s Disease. Biol. Psychiatry.

[71] Tan X (2022). MicroRNA-409-3p targeting at ATXN3 reduces the apoptosis of dopamine neurons based on the profile of miRNAs in the cerebrospinal fluid of early Parkinson’s Disease. Front. Cell Dev. Biol..

[72] Tong, G., Zhang, P., Hu, W., Zhang, K., & Chen, X. Diagnostic test to identify Parkinson’s Disease from the blood sera of Chinese population: A cross-sectional study. *Parkinson’s Dis.* **2022**, Article ID 8683877 (2022). 10.1155/2022/8683877 PMC9007633 35432916

[73] Wang J, Chen C, Zhang Y (2020). An investigation of microRNA-103 and microRNA-107 as potential blood-based biomarkers for disease risk and progression of Alzheimer's Disease. J. Clin. Lab. Anal..

[74] Xing R, Li L, Liu X, Tian B, Cheng Y (2020). Down regulation of miR -218, miR -124, and miR -144 relates to Parkinson’s disease via activating NF-κB signaling. Kaohsiung J. Med. Sci..

[75] Yan JH (2020). Identification of microRNAs for the early diagnosis of Parkisnon's disease and multiple system atrophy. J. Integr. Neurosci..

[76] Yang TT, Liu CG, Gao SC, Zhang Y, Wang P-C (2018). The serum exosome derived microRNA-135a, -193b, and -384 were potential Alzheimer’s Disease biomarkers. Biomed. Environ. Sci..

[77] Yang Z (2019). Altered expression levels of microRNA-132 and Nurr1 in peripheral blood of Parkinson’s disease: a potential disease biomarker. ACS Chem. Neurosci..

[78] Yang Z (2019). Elevated plasma microRNA-105-5p level in patients with idiopathic Parkinson’s disease: A potential disease biomarker. Front. Neurosci..

[79] Yang P, Lin G, Wang M, Chen X, Hua J (2022). Long non-coding RNA ANRIL interacts with microRNA-34a and microRNA-125a, and they all correlate with disease risk and severity of Parkinson's disease. JCLA..

[80] Yao Y, Qu M, Li G, Zhang F, Rui H (2018). Circulating exosomal miRNAs as diagnostic biomarkers in Parkinson’s disease. Eur. Rev. Med. Pharmacol. Sci.

[81] Zhang L, Zhang J, Wang K, Wang R (2020). Serum microRNA-30c-5p and microRNA-373 expressions as potential biomarkers for Parkinson’s disease. All Life.

[82] Zhao N, Jin L, Fei G, Zheng Z, Zhong C (2014). Serum microRNA-133b is associated with low ceruloplasmin levels in Parkinson’s disease. Parkinsonism Relat. Disord..

[83] Zhou Y (2016). MicroRNA-7 targets Nod-like receptor protein 3 inflammasome to modulate neuroinflammation in the pathogenesis of Parkinson’s disease. Mol. Neurodegener..

[84] Zhu, J., Xu, X., Liang, Y., & Zhu, R. Downregulation of microRNA-15b-5p targeting the Akt3- Mediated GSK-3β/β-Catenin signaling pathway inhibits cell apoptosis in Parkinson’s Disease. *BioMed. Res*. Int. **2021**, Article ID 8814862 (2021), 10.1155/2021/8814862 PMC7806375 33506036

[85] Bissonnette S (2018). MicroRNAs as biomarkers for Parkinson’s disease. Movement Disord..

[86] Briggs CE (2015). Midbrain dopamine neurons in Parkinson’s disease exhibit a dysregulated miRNA and target-gene network. Brain. Res..

[87] Burgos K (2014). Profiles of extracellular miRNA in cerebrospinal fluid and serum from patients with Alzheimer’s and Parkinson’s Diseases correlate with disease status and features of pathology. PLoS ONE.

[88] Cho HJ (2013). MicroRNA-205 regulates the expression of Parkinson’s disease-related leucine-rich repeat kinase 2 protein. Hum. Mol. Genet..

[89] Kim J (2007). A MicroRNA feedback circuit in midbrain dopamine neurons. Science.

[90] Nair VD, Ge Y (2016). Alterations of miRNAs reveal a dysregulated molecular regulatory network in Parkinson’s disease striatum. Neurosci. Lett..

[91] Patil KS (2019). Combinatory microRNA serum signatures as classifiers of Parkinson’s disease. Parkinsonism Relat. Disord..

[92] Sethi P, Lukiw WJ (2009). Micro-RNA abundance and stability in human brain: Specific alterations in Alzheimer’s disease temporal lobe neocortex. Neurosci. Lett..

[93] Thomas R, Keeney P, Bennett J (2012). Impaired complex-I mitochondrial biogenesis in Parkinson disease frontal cortex. J. Parkinsons Dis..

[94] Alvarez-Erviti L (2010). Chaperone-mediated autophagy markers in Parkinson disease brains. Arch. Neurol..

[95] Cardo LF (2013). Profile of microRNAs in the plasma of Parkinson’s disease patients and healthy controls. J. Neurol..

[96] Cardo LF (2014). MiRNA profile in the substantia nigra of Parkinson’s Disease and healthy subjects. J. Mol. Neurosci..

[97] Cosín-Tomás M (2017). Plasma miR-34a-5p and miR-545-3p as early biomarkers of Alzheimer’s disease: Potential and limitations. Mol. Neurobiol..

[98] Fernández-Santiago R (2015). MicroRNA association with synucleinopathy conversion in rapid eye movement behavior disorder. Ann. Neurol..

[99] Miñones-Moyano E (2011). MicroRNA profiling of Parkinson’s disease brains identifies early downregulation of miR-34b/c which modulate mitochondrial function. Hum. Mol. Genet..

[100] Pérez-Soriano, A. *et al*. MicroRNA deregulation in blood serum identifies multiple system atrophy altered pathways. *Move. Disord*. **35**, No. 10 (2020). 10.1002/mds.28143 32687224

[101] Tolosa E (2018). MicroRNA alterations in iPSC-derived dopaminergic neurons from Parkinson disease patients. Neurobiol. Aging.

[102] Villar-Menéndez I (2014). Increased striatal adenosine A2A receptor levels is an early event in Parkinson’s disease-related pathology and it is potentially regulated by miR-34b. Neurobiol. Dis..

[103] Caggiu E (2018). Differential expression of miRNA 155 and miRNA 146a in Parkinson’s disease patients. eNeurologicalSci..

[104] Grossi I (2021). MicroRNA-34a-5p expression in the plasma and in its extracellular vesicle fractions in subjects with Parkinson's disease: An exploratory study. Int. J. Mol. Med..

[105] Schwienbacher C (2017). Plasma and white blood cells show different miRNA expression profiles in Parkinson’s disease. J. Mol. Neurosci..

[106] Serafin A (2015). Overexpression of blood microRNAs 103a, 30b, and 29a in L-dopa-treated patients with PD. Neurology.

[107] Vallelunga A (2014). Identification of circulating microRNAs for the differential diagnosis of Parkinson’s disease and Multiple System Atrophy. Front. Cell Neurosci..

[108] Vallelunga A (2019). Serum miR-30c-5p is a potential biomarker for multiple system atrophy. Mol. Biol. Rep..

[109] Vallelunga A (2021). Serum miR-96-5P and miR-339-5P are potential biomarkers for multiple system atrophy and Parkinson's Disease. Front. Aging Neurosci..

[110] Zago E (2022). Early downregulation of hsa-miR-144-3p in serum from drug-naïve Parkinson’s disease patients. Sci. Rep..

[111] Caldi Gomes L (2022). Multi-omic landscaping of human midbrains identifies disease-relevant molecular targets and pathways in advanced-stage Parkinson’s disease. Clin. Transl. Med..

[112] Kurz A (2021). Differential expression of gut miRNAs in idiopathic Parkinson’s disease. Parkinsonism Rel. Disord..

[113] Schlaudraff F (2014). Orchestrated increase of dopamine and PARK mRNAs but not miR-133b in dopamine neurons in Parkinson’s disease. Neurobiol. Aging.

[114] Tatura R (2016). Parkinson’s disease: SNCA-, PARK2-, and LRRK2- targeting microRNAs elevated in cingulate gyrus. Parkinsonism Relat. Disord..

[115] Baghi M (2020). Modified level of miR-376a is associated with Parkinson’s disease. J. Cell Mol. Med..

[116] Baghi M (2021). MiR-193b deregulation is associated with Parkinson's disease. J. Cell Mol. Med..

[117] Behbahanipour M (2019). Expression profiling of blood microRNAs 885, 361, and 17 in the patients with the Parkinson’s disease: Integrating interaction data to uncover the possible triggering age-related mechanisms. Sci. Rep..

[118] Yousefi M, Peymani M, Ghaedi K, Irani S, Etemadifar M (2022). Significant modulations of linc001128 and linc0938 with miR-24-3p and miR-30c-5p in Parkinson disease. Sci. Rep..

[119] Takahashi I (2015). Identification of plasma microRNAs as a biomarker of sporadic Amyotrophic Lateral Sclerosis. Mol. Brain.

[120] Uwatoko H (2019). Identification of plasma microRNA expression changes in multiple system atrophy and Parkinson’s disease. Mol. Brain.

[121] Martins M (2011). Convergence of miRNA expression profiling, α-synuclein interaction and GWAS in Parkinson’s Disease. PLoS ONE.

[122] Oliveira SR (2020). Circulating inflammatory miRNAs associated with Parkinson’s disease pathophysiology. Biomolecules.

[123] Yılmaz ŞG (2016). Hypothesis: do miRNAs targeting the leucine-rich repeat kinase 2 gene ( *LRRK2*) influence Parkinson’s Disease susceptibility?. OMICS.

[124] Alieva AKh (2015). miRNA expression is highly sensitive to a drug therapy in Parkinson’s disease. Parkinsonism Relat. Disord..

[125] Starhof C (2018). The biomarker potential of cell-free microRNA from cerebrospinal fluid in Parkinsonian syndromes. Movement Disord..

[126] Chiu C-C (2019). Upregulated expression of microRNA-204-5p leads to the death of dopaminergic cells by targeting DYRK1A-mediated apoptotic signaling cascade. Front. Cell Neurosci..

[127] McMillan KJ (2017). Loss of microRNA-7 regulation leads to α-synuclein accumulation and dopaminergic neuronal loss *in vivo*. Mol. Ther..

[128] Ghit A, El Deeb H (2022). Cytokines, miRNAs, and antioxidants as combined non-invasive biomarkers for Parkinson’s disease. J. Mol. Neurosci..

[129] Sulaiman SA (2020). Differential expression of circulating miRNAs in Parkinson's disease patients: Potential early biomarker?. Neurology Asia..

[130] Hoehn MM, Yahr MD (1967). Parkinsonism: onset, progression, and mortality. Neurology.

[131] Warnecke, T., Schäfer, K.H., Claus, I., Del Tredici, K., & Jost, W.H. Gastrointestinal involvement in Parkinson's disease: pathophysiology, diagnosis, and management. *NPJ Parkinsons Dis.* **24**, 81:31 (2022). 10.1038/s41531-022-00295-x PMC8948218 35332158

[132] Chis AR (2021). Plasma hsa-mir-19b is a potential Levodopa therapy marker. J. Cell Mol. Med..

[133] Yuan Q (2020). Comprehensive analysis of core genes and key pathways in Parkinson's disease. Am. J. Transl. Res..

[134] Long H-Z (2021). PI3K/AKT signal pathway: A target of natural products in the prevention and treatment of Alzheimer's disease and Parkinson's disease. Front. Pharmacol..

[135] Kook S (2020). Mdm2 enhances ligase activity of parkin and facilitates mitophagy. Sci. Rep..

[136] Deeks JJ, Macaskill P, Irwig L (2005). The performance of tests of publication bias and other sample size effects in systematic reviews of diagnostic test accuracy was assessed. J. Clin. Epidemiol..

[137] Georgiev, D., Hamberg, K., Hariz, M., Forsgren, L., & Hariz, G-M. Gender differences in Parkinson's disease: A clinical perspective. *Acta Neurol. Scand*. 1–15 (2017). 10.1111/ane.12796 28670681

[138] Rich-Edwards JW, Kaiser UB, Chen GL, Manson JE, Goldstein JM (2018). Sex and gender differences research design for basic, clinical, and population studies: Essentials for investigators. Endo. Rev..

[139] Durcan TM, Fon EA (2015). The three 'P's of mitophagy: PARKIN, PINK1, and post-translational modifications. Genes Dev..

[140] Valentin-Vega YA, Kastan MB (2012). A new role for ATM. Autophagy.

[141] O’Flanagan CH, Morais VA, Wurst W, De Strooper B, O’Neill C (2015). The Parkinson’s gene PINK1 regulates cell cycle progression and promotes cancer-associated phenotypes. Oncogene.

[142] Maynard S, Fang EF, Scheibye-Knudsen M, Croteau DL, Bohr VA (2015). DNA damage, DNA repair, aging, and neurodegeneration. Cold Spring Harb. Perspect. Med..

[143] Reeve A, Simcox E, Turnbull D (2014). Ageing and Parkinson’s disease: Why is advancing age the biggest risk factor?. Ageing Res. Rev..

[144] Tompkins MM, Basgall EJ, Zamrini E, Hill WD (1997). Apoptotic-like changes in Lewy-body-associated disorders and normal aging in substantia nigral neurons. Am. J. Pathol..

[145] Liu W, Geng L, Chen Y (2018). MiR-19b alleviates MPP^+^-induced neuronal cytotoxicity *via* targeting the HAPLN4/MAPK pathway in SH-SY5Y cells. RSC Adv..

[146] Yang H, Zhang L, Wang Q (2021). MicroRNA-221-3p alleviates cell apoptosis and inflammatory response by targeting cyclin dependent kinase inhibitor 1B in chronic obstructive pulmonary disease. Bioengineered.

[147] Zhou Y (2023). *CircEPS15*, as a sponge of *MIR24-3p* ameliorates neuronal damage in Parkinson disease through boosting PINK1-PRKN-mediated mitophagy. Autophagy.

[148] Wang ZH (2015). MicroRNA-214 participates in the neuroprotective effect of Resveratrol via inhibiting α-synuclein expression in MPTP-induced Parkinson's disease mouse. Biomed Pharmacother..

[149] Ho PTB, Clark IM, Le LTT (2022). MicroRNA-based diagnosis and therapy. Int. J. Mol. Sci..

[150] McDonald JS, Milosevic D, Reddi HV, Grebe SK, Algeciras-Schimnich A (2011). Analysis of circulating microRNA: Preanalytical and analytical challenges. Clin. Chem..

[151] Blondal T (2013). Assessing sample and miRNA profile quality in serum and plasma or other biofluids. Methods.

[152] Mitchell PS (2008). Circulating microRNAs as stable blood-based markers for cancer detection. Proc. Nat. Acad. Sci..

[153] Wang K (2012). Comparing the microRNA spectrum between serum and plasma. PLoS ONE.

[154] Coenen-Stass AML (2018). Evaluation of methodologies for microRNA biomarker detection by next generation sequencing. RNA Biol..

[155] Tylee DS, Kawaguchi DM, Glatt SJ (2013). On the outside, looking in A review and evaluation of the comparability of blood and brain “-omes”. Am. J. Med. Genet. Part B Neuropsych. Genet..

[156] McMillan KJ (2017). Loss of microRNA-7 regulation leads to α-synuclein accumulation and dopaminergic neuronal loss in vivo. Mol. Ther..

