# Supplementary material for: MicroRNAs in Parkinson’s disease: a systematic review and diagnostic accuracy meta-analysis
Source: Sci Rep. 2023 Sep 28;13:16272. doi: 10.1038/s41598-023-43096-9 (PMC10539377; doi:10.1038/s41598-023-43096-9)
Supplement: Supplementary file 1 — Supplementary Information. [file 41598_2023_43096_MOESM1_ESM.pdf]

MicroRNAs in Parkinson’s disease: A systematic review and diagnostic accuracy meta-analysis

Diane Guévremont, Joyeeta Roy, Nicholas J. Cutfield, Joanna M. Williams.

Supplementary Table S1. Characteristics of studies included in the systematic review and meta-analysis on microRNAs in Parkinson’s disease patients compared to controls. BBC – brain bank criteria; CT – controls; DA – dopamine; IRDB – idiopathic REM sleep behaviour disorder, LEDD – levodopa equivalent daily dose; MDS – Movement Disorders Society Clinical Diagnostic Criteria, NP – neuropathology; PD – Parkinson’s disease; STN-DBS – subthalamic nucleus deep brain stimulation; UKPDSBBC = UK Parkinson’s Disease Brain Bank Criteria; † newly diagnosed PD patients; *median value; **mean ± SEM.

Study	Ref	Country	# PD	# CT	# Men (PD)	# Men (CT)	Age PD (Years ± SD)	Age HC (Years ± SD)	Disease Duration (Y ± SD)	H&Y Score (score ± SD or range)	Diagnostic Criteria for PD	PD Type	PD Medication Status (n=)	PD Medication Type and Dose (mg/day) or LEDD (mg/day ± SD)
**Alieva 2015**	**124**	Russia	38	24	19	12	55.6 ± 7.6	-	-	1–2	UKPDSBBC	Mixed	Treated (18); Untreated (20)	Pramipexole 1.5; Piribedil 150; Levodopa 150-200; Amantadine 300
**Alvarez-Erviti 2013**	**94**	Spain	6	5	-	-	-	-	-	-	Queen Square BBC	Idiopathic	-	-
**Baghi 2020**	**115**	Iran	33	25	23	16	62.9 ± 11.4	60.3 ± 10.1	5.8 ± 4.9	1–4		-	-	-
**Baghi 2021**	**116**	Iran	20	20	12	14	61.7 ± 12.6	58.45 ± 9.4	7.39 ± 5.71	2.3 ± 0.95 (1-4)	-	-	-	-
**Bai 2017**	**58**	China	80	80	48	48	64.0 ± 5.8	63.3 ± 5.4	4.4 ± 4.4	1–3	UKPDSBBC	Idiopathic	Levodopa Treated (61); Untreated (19)	312.4 ± 319.2
**Barbagallo 2020**	**29**	Italy	30	30	24	10	69.6 ± 8.0	67.9 ± 8.2	6.9 ± 3.6	2.6 ± 0.9	Gelb	-	Treated	-
**Behbahanipour 2019**	**117**	Iran	36	16	25	11	61.3 ± 11.4	62.5 ± 12.4	5.8 ± 5.6	1–4	UKPDSBBC	-	-	-
			Early stage (28)	-	20	-	60.9 ± 10.6	-	4.9 ± 4.8	1–2				
			Advanced stage (8)		5		62.8 ± 14.6		9.3 ± 7.4	3–4				

**Bissonnette 2018**	**85**	USA	10	10	-	-	-	-	-	-	UKPDSBBC	-	-	-
**Botta-Orfila 2014**	**30**	Spain	Pilot study (20)	10	10	5	61.1 ± 10.1	65 ± 9.1	-	-	UKPDSBBC	Idiopathic, LRRK2	-	-
			Validation 1 (20)	20	10	10	69.75 ± 1	71.3 ± 8.4				Idiopathic, LRRK2		
			Validation 2 (65)	65	29	29	68.1 ± 10	66.3 ± 11.4	9.6	1–5		Idiopathic		
**Briggs 2015**	**86**	USA	8	8	5	5	-	-	-	-	UKPDSBBC	Idiopathic	-	-
**Burgos 2014**	**87**	USA	CSF (65)	70	-	-	80.0 ± 5.1	82.1 ± 10	12.6 ± 7.9	-	-	-	-	-
			Serum (60)	72										
**Caggiu 2018**	**103**	Italy	37	43	7	18	71.3 ± 9.6	60.0 ± 13.1	8.3 ± 4.3	3.3 ± 1.2	Gelb	-	Treated	458.3 ± 227.7
**Caldi Gomes 2022**	**111**	Germany	Discovery (13)	10	8	4	78.3 ± 1.3	77.1 ± 3.4	13.4 ± 1.3	-	-	Idiopathic	-	-
			Replicatio n (6)	2	12	0	82.7 ± 1.7	74.5 ± 5.3	12.2 ± 1					
**Cao 2017**	**31**	China	109	40	73	25	59.9 ± 13.3	67.9 ± 8.6	9.2 ± 7.4	1–5	UKPDSBBC	-	-	-
**Cardo 2013**	**95**	Spain	31	25	17	13	63.9 ± 11.9	67.6 ± 15.2	†	-	UKPDSBBC	-	Untreated	-
**Cardo 2014**	**96**	Spain	8	4	3	2	77.4 ± 9.0	69.0 ± 11.7	-	-	LBs and SN neuron loss	Idiopathic	-	-
**Chen 2016**	**59**	China	24	61	14	34	58.51 ± 5.6	54.2 ± 12.8	2.4 ± 1.1	-	UKPDSBBC		-	-
**Chen 2017**	**45**	China	169	170	81	91	61.9 ± 5.1	61.6 ± 3.3	5.8 ± 4.2	2.0 ± 0.8	UKPDSBBC	Sporadic	Treated	500.7 ± 350.5
**Chen 2018**	**60**	China	25	25	16	16	65.0 ± 8.7	-	†	-	UKPDSBBC	-	Untreated	
**Chen 2020**	**46**	China	30	30	20	16	63.2 ±10.2	59.6 ± 12.8	-	2	MDS-UPDRS	-	-	-
**Chen 2021**	**32**	China	46	45	13	18	63.09 (60.2–66)	61.54 (60–63.1)	5.72 (4.43–7.01)	2.86 (2.57–3.15)	MDS-UPDRS	-	-	-
**Chis 2021**	**132**	Romania	Discovery (10)	10	6	6	65.9 (6.1)	68.1 (6.4)	-	1.9 (0.7)	MDS	-	treated	-
			Validation (66)	29	35	14	68 (9.1)	63.5 (9.1)	-	2.5 (0.8)	MDS	-	treated	-
			Validation (10 untreated/1 0 treated)	-	7	-	67.2 (6.6)	-	-	-	-	-	Untreated/ Treated	-
**Chiu 2019**	**126**	Taiwan	50	50	24	23	66.4 ± 1.0	63.7 ± 1.1	-	-	Gelb	Sporadic	-	-
**Cho 2013**	**88**	USA	15	11	-	-	80.0 ± 6.9	85 ± 6.6	-	-	LBs and SN neuron loss	Sporadic	-	-

**Cosín-Tomás 2017**	**97**	Spain	21	21	-	-	-	69.6 (67.2 - 71.9)	-	-	-	-	-	-
**Cressatti 2020**	**47**	Canada	84	83	49	39	71.4 ± 12.7	67.3 ± 9.5	-	1–3+	UKPDSBBC	-	Treated	658.3 ± 312.6
**Ding 2016**	**33**	China	106	91	61	45	60.1 ± 12.2	60.7 ± 9.7	-	1–5	-	-	-	-
**Dong 2016**	**34**	China	122	104	62	51	67.6 ± 7.5	66.0 ± 5.3	-	1–5	-	-	-	-
**Dos Santos 2018**	**35**	Germany	40	40	20	20	61.0 ± 1.0	64.0 ± 1.0	1.8 ± 1.0	2*	UKPDSBBC	Akinetic-rigid	-	-
**Fazeli 2020**	**36**	Iran	30	14	21	11	62 ±11.1	63.93 ± 12	3-11	1-3	MDS-UPDRS	-	-	-
**Fernández-Santiago 2015**	**98**	Spain	8	28	6	25	74.4 ± 4.4	70.9 ± 5.8	-	-	UKPDSBBC	IRDB to PD	-	-
**Fu 2017**	**9**	China	15	15	-	-	-	-	-	-	-	-	-	-
**Ghit 2022**	**128**	Egypt	20	15	11	10	61.7 ± 14.6	61.8 ± 4.9	4.3 ± 4.0	-	UKPDSBBC	Idiopathic	-	-
**Gong 2022**	**61**	China	5	5	3	3	55.8 ± 7.1	59.8 ± 8.1	-	-	MDS	-	-	-
**Grossi 2021**	**104**	Italy	15	14	15	14	75.7 ± 3.0	78.5 ± 7.3	2.2 ± 0.6	-	UKPDSBBC	-	Treated (15)	312.5 ± 236.6
**Gui 2015**	**62**	China	Discovery (47)	27	25	9	63.0 ± 9.0	60.0 ± 13.0	11.5 ± 6.6	-	UKPDSBBC	Sporadic	-	-
			Validation (78)	35	41	-	64.0 ± 12.0	-	-	-				
**Han 2020**	**63**	China	39	40	22	23	61.5 ± 5.5	63.8 ± 5.6	2.7 ± 2.4	1.9 ± 0.9	-	-	-	390.0 ± 204.7
**He 2021**	**64**	China	Stage II (8); Stage III (42); Stage IV (22)	31	Stage II (5); Stage III (26); Stage IV (12)	17	59.8 ± 7.6 (Stage II); 61.6 ± 7.6 (Stage III); 64.7 ± 8.1 (Stage IV)	63.9 ± 7.4	-	-	UKPDSBBC	Idiopathic	-	-
			Stage II (7); Stage III (12); Stage IV (11)	10	-	-	-	63.9 ± 7.4	-	-				
**Hoss 2016**	**4**	USA	29	33	29	33	77.6 ± 9.0	68.1 ± 14.8	10.5 ± 6.5	-	-	-	-	-
**Jiang 2021**	**37**	China	50	30	19	14	63.6 ± 11.7	59.7 ± 11.2	3.7 ± 3	2.1 ± 0.8	MDS-UPDRS	Idiopathic	Treated (28)	LEDD (mg) 325.00 ± 297.07
			Screening phase (5)	5	-	-	-	-	-	-			-	-
			Validation 1 (30)	30	-	-	-	-	-	-			-	-
			Validation 2 (50)	30	-	-	-	-	-	-			-	-

**Jin 2018**	**10**	China	46	46	23	25	60.2 ± 9.9	61.3 ± 9.0	-	1–3	UKPDSBBC	Tremor-dominant, bradykinesia, rigidity-dominant, mixed	-	-
**Khoo 2012**	**48**	USA	Discovery (32)	32	16	15	66 ± 11 (M); 67 ± 11 (F)	65 ± 10 (M); 62 ± 17 (F)	-	1.8 ± 2.3 (M); 1.5 ± 2.4 (F)	UKPDSBBC	Idiopathic	10 non-medicated, 20 medicated	-
			Replication (42)	30	20	10	68 ± 6 (M); 72 ± 8 (F)	64 ± 15 (M); 59 ± 14 (F)	-	1.8 ± 1.8 (M); 1.6 ± 1.5 (F)				
			Validation (30)	8	16	3	68 ± 10 (M); 71 ± 7 (F)	71 ± 3 (M); 73 ± 4 (F)	-	2.1 ± 0.7 (M); 2.4 ± 0.6 (F)				
**Kim 2007**	**89**	USA	3	5	3	5	70.0 ± 8.9	79.0 ± 12.5	2.6 ± 1.2	-	UKPDSBBC	-	-	-
**Kurz 2021**	**112**	Germany	13	17	10	9	70.4 ± 6.9	64.9 ± 8.9	-	2.5 ± 1.4	UKPDSBBC	Idiopathic	-	-
**Li 2017**	**49**	China	60	60	33	30	68.3 ± 7.5	67.0 ± 7.6	-	-	UKPDSBBC	Sporadic idiopathic	-	-
			PD-Dep (24)		16		68.96 ± 5.9		5.22 ± 4.1	3.08 ± 1.1				
			PD-NDep (36)		17		67.92 ± 8.9		3.83 ± 4.2	2.19 ± 1.2				
**Li 2020**	**50**	China	80	60	42	31	64.6 ± 7.5	64.0 ± 7.3	4.8 ± 3.23	2.2 ± 0.8	UKPDSBBC	-	-	-
**Li 2021**	**51**	China	pPD (25)	21	13	10	68 (63 - 70)	64 (62 - 66)	-	-	UKPDSBBC	-	-	-
			dnPD (20)		9		65 (64 - 68)		1.5 (1 - 2.75)	2 (1.13 - 2)		-	not treated	-
			aPD (24)		12		66.5 (63.3 - 69)		8 (7 - 9)	2.5 (2.5 - 3)		-	treated	-
**Lin 2022**	**65**	China	92	64	-	-	-	-	-	-	UKPDSBBC	-	-	-
**Ma 2016**	**66**	China	138	112	75	61	29.4 ± 13.3	31.2 ± 19.2	-	1–5	UKPDSBBC	-	-	-
**Manna 2021**	**38**	Italy	45	39	26	15	64 ± 8	63.7 ± 7.5	5.57 ± 4.1	2 (1–4)	MDS-UPDRS	-	Treated	-
			Discovery (5)	5			71 ± 5.41	69 ± 4.38	-	-				
			Validation (40)	34			66 ± 9	64 ± 8	-	-				
**Margis 2011**	**8**	Brazil	Untreated (8)	8	4	4	66 ± 6.7	67.0 ± 8.0	3.0 ± 2.6	1–2	UKPDSBBC	-	Untreated (8)	-
			Early onset (7)		4		45 ± 8.7	67 ± 8	7.2 ± 6.6	1–3			Early-onset treated (7)	Levodopa/ Carbidopa
**Marques 2017**	**39**	Netherlands	28	28	21	15	54.5 ± 10.4	62.9 ± 8	3.2 ± 3.4	1.8 ± 0.6	UKPDSBBC	Sporadic	-	-

**Martins 2011**	**121**	Portugal	19	13	10	5	65.1 ± 4.4	64.4 ± 5.9	8.7 ± 5.1	2.6 ± 0.8	UKPDSBBC	Idiopathic	-	-
**McMillan 2017**	**127**	UK	6	5	2	1	83.0 ± 4.0	78 ± 4.7	16.2 ± 5.9	-	-	-	Treated	Levodopa
**Miñones-Moyano 2011**	**99**	Spain	Treated (16)	28	10	17	74.6 ± 11.0	58.6 ± 16.0	-	-	-	Braak Stages 4-5	Treated	-
			Untreated (7)		5		68.1 ± 16.3		-	-	-	Braak Stages 1-3 (undiagnosed)	Untreated	-
**Mo 2017**	**53**	China	44	42	26	24	56.8 ± 17.1	55.4 ± 12.5	2.2 ± 1.3	1–2.5	UKPDSBBC	-	-	-
**Nair 2016**	**90**	USA	12	12	6	6	75.6 ± 8.4	74.1 ± 11.6	-	-	-	-	Treated (10)	Levodopa
**Nie 2020**	**67**	China	7	34	1	10	61.9 ± 8.4	33.2 ± 9.6	-	-	UKPDSBBC	-	-	-
**Oliveira 2020**	**122**	Portugal	Discovery (20)	20	10	10	71.6 ± 9.2	69.5 ± 8.1	11.9 ± 8.9	2.3 ± 0.6	MDS	Idiopathic	Treated	All patients were taking antiparkinsonic medication
			Validation (20)	20	10	10	69.2 ± 11.3	65.3 ± 8.3	12.6 ± 9.4	2.6 ± 1.1	MDS	Idiopathic	Treated	All patients were taking antiparkinsonic medication
**Ozdilek 2020**	**54**	Turkey	51	20	31	12	64.0 ± 9.0	58.6 ± 7.1	5.7 ± 3.8	2.0 ± 0.7	UKPDSBBC	-	Treated	938.7 ± 507.6
**Patil 2019**	**91**	USA/Norway/ Sweden	Pilot (16)	8	8	4	65.8 ± 6.9	66.9 ± 8.9	-	1.7 ± 0.3	UKPDSBBC	-	Untreated, newly diagnosed	-
			Verificatio n (164)	182	101	93	68.3 ± 9.4	66.5 ± 9.5	-	1.9 ± 0.6				
			Validation (42)	22	21	12	68.5 ± 9.8	68.3 ± 7.0	-	-				
**Perez-Soriano 2020**	**100**	Spain	Discovery (19)	40	12	28	64 ± 12	66 ± 11.5	5.3 ± 2.9	1.6 ± 0.5	-	-	Treated	511 ± 278
			Validation (18)	20	8	10	64 ± 8.6	64.5 ± 9.6	5.4 ± 2.3	2.1± 0.6	-	-	Treated	630 ± 320
**Qin 2019**	**68**	China	20	27	10	14	66.5 ± 9.0	63.7 ± 7.9	1.99 ±1.01	-	UKPDSBBC	Sporadic	-	-
**Ravanidis 2019; 2020**	**41**	Greece	Initial study (99 IPD)	101	55	23	67.1 ± 12.4	61.6 ± 10.6	7.3 ± 7.2	-	MDS	Idiopathic	-	-
			Validation (109 iPD)	92	57	33	64.2 ± 10.4	57.1 ± 12	5.4 ± 5.8	-			-	-
			Pooled (208 iPd)	193	112	56	-	-	-	-			-	-
**Schlaudraff 2014**	**113**	Germany	5	8	3	4	78.2 ± 1.3	69 ± 1.6	-	-	Sporadic	Braak Stages 0–5	-	-
**Schwienbacher 2017**	**105**	Italy	L-dopa set 1 (50)	49	26	26	65.2 ± 10.4	65.2 ± 10.0	6.7 ± 5.0	1–3	Gelb	-	Treated Set 1	Levodopa
			L-dopa set 2 (50)	49	23	23	68.1 ± 8.9	68.6 ± 8.7	6.9 ± 5.3	1–4		-	Treated Set 2	
			Drug naïve (10)	10	6	4	67.5 ± 6.9	67.1 ± 7.3	3.8 ± 3.2	1–2.5		-	Untreated	-

**Serafin 2015**	**106**	Italy	l-Dopa (36)	36	-	-	-	-	-	-	Gelb	-	Treated	-
			Drug naïve (10)	10	-	-	-	-	-	-		-	Untreated	-
**Sethi 2009**	**92**	USA	4	6	-	-	69.5 ± 1.7	69.0 ± 1.8	-	-	Idiopathic	-	-	-
**Sheinerman 2017**	**42**	USA	50	50	35	24	66.76 ± 7.7	64.1 ± 9.8	10 ± 5.1	-	Queen Square BBC	-	-	-
**Shu 2020**	**69**	China	82	44	52	27	68.5 ± 7.5	66.2 ± 8.6	-	2.47 ± 0.64	UPDRS III, V and MRI	-	-	-
**Soreq 2013**	**56**	Israel	7	6	7	6	56 ± 4.0	56.5 ± 6.2	-	-	-	-	Treated and STN-DBS	-
**Starhof 2018**	**125**	Denmark	37	23	25	11	66.3 ± 12.0	41.5 ± 17.6	6.9 ± 3.8	2*	UKPDSBBC	-	Treated	536.6 ± 413.1
**Su 2019**	**70**	China	28	28	-	-	-	-	-	1	UKPDSBBC	Idiopathic	-	-
**Sulaiman 2020**	**129**	Malaysia	EOPD (14); LOPD (14)	11	11	3	EOPD (53.9 ± 1.8); LOPD (68.3 ±1.3)	(37 ± 2.3); (54.8 ±1.5)	< 10 years 17<br>> 10 years 11	-	UKPDSBBC	Idiopathic	Treated (28)	-
**Takahashi 2015**	**119**	Japan	30	47	17	29	69.0 ± 5.9	67.1 ± 10.9	-	-	-	-	-	-
**Tan 2022**	**71**	China	7	4	3	1	53 ±5	46 ± 10	2.5 ± 1.5 (early PD)	2 ± 0.7	MDS-UPDRS	-	-	-
**Tatura 2016**	**114**	Germany	22	10	10	4	73.9 ± 6.9	65.7 ± 10.9	-	-	NP	T limbic (8); neocortical (10)	-	-
**Thomas 2012**	**93**	USA	8	8	-	-	-	-	-	-	-	-	-	-
**Tolosa 2018**	**101**	Spain	sPD (3)	4	0	2	60.0 ± 7.9	53.3 ± 8.8	-	-	-	Sporadic	-	-
			L2PD (3)		1		58.3 ± 12.7		-	-	-	LRRK2	-	-
**Tong 2022**	**72**	China	209	50	115	29	68.2 ± 5.4	65.6 ± 4.3	-	-	-	-	-	-
**Uwatoko 2019**	**120**	Japan	28	28	13	15	69 ± 1.32	63.2 ± 1.3	9.6 ± 1.0	-	-	-	-	-
**Vallelunga 2014**	**107**	Italy	25	25	13	13	between 46 and 60	between 46 and 60	< 6	3.0 ± 0.0	UKPDSBBC	-	Treated	730.5 ± 392.8
**Vallelunga 2019**	**108**	Italy	56	50	37	23	62.1 ± 6.9	64.6 ± 12.5	2.2 ± 2.3	-	UKPDSBBC	-	-	-
**Vallelunga 2021**	**109**	Italy	51	56	29	25	61 ± 7	63 ± 12	2 ± 2.4	-	UKPDSBBC	-	Treated	302.5 ± 82
**Villar-Menéndez 2014**	**102**	Spain	Braak Stages 1-2 (6)	26	4	17	78.8 ± 12.1	56.9 ± 12.7	-	-	NP	Braak Stages 1-2	-	-
			Braak Stages 3-4 (13)		7		72.3 ± 10.2		-	-		Braak Stages 3-4	-	-
			Braak Stage 5 (6)		4		79.7 ± 6.2		-	-		Braak Stage 5	-	-

**Wake 2016**	**57**	USA	29	36	29	33	77.6 ± 7.8	68.6 ± 14.3	-	-	-	-	-	-
**Wang 2020**	**73**	China	120	120	64	54	70.5 ± 8.3	71.2 ± 10.8	-	-	Parkinson's Disease Society Brain Bank	-	-	-
**Wu 2020**	**43**	China	85	50	41	27	63.5 ± 7.4	61.2 ± 5.9	-	I 24 (28.24) - II 33 (38.82) - III 28 (32.94)	MDS-ES-EFNS	-	Treated (85) Levodopa	start 250mg, 2-4 times/d. then Up to125-750mg every 3-7 days, 4-6 times/d, for 12 weeks of
**Xing 2020**	**74**	China	15	10	9	5	70.1 ± 7.1	70.6 ± 7.6	5.9 ± 5.1	3.2 ± 0.8	-	-	-	-
**Yan 2020**	**75**	China	23	24	10	12	59.3 ± 8.8	62.8 ± 5.7	1	2.1 ± 0.8	UKPDSBBC	-	All Untreated	patients did not take anti-PD drugs
**Yang 2018**	**76**	China	30	30	11	-	68.6 ± 5.5	-	-	-	MDS	-	-	-
**Yang 2019 A**	**77**	China	319	273	176	147	67.6 ± 9.6**	68.3 ± 9.6**	-	-	MDS	Idiopathic	Untreated (80); Treated (91); Treated (levodopa+ DA agonists – 85)	-
**Yang 2019 B**	**78**	China	269	222	157	130	66.1 ± 0.6	66.2 ± 0.6	-	1–3	MDS	Sporadic	Treated – dopamine agonist (13); levodopa (34); Untreated (46)	-
**Yang 2021**	**79**	China	78	78	55	48	65.9 ± 5.6	64.6 ± 5.2	-	-	MDS	Incipient	Treated	-
**Yao 2018**	**80**	China	52	48	28	26	65.6 ± 10.5	61.2 ± 9.0	5.3 ± 1.6	-	UKPDSBBC	-	Treated	Levodopa/carbidopa (52); Entacapone (20); Selegiline (10); Ropinirole (9); Pramipexole (5); Amantadine (4)
**Yilmaz 2016**	**123**	Turkey	102	102	65	44	67.1 ± 1.1	62.7 ± 0.9	-	-	-	-	Treated	-
**Yousefi 2022**	**118**	Iran	38	20	21	10	64.4 ± 11	59.4 ± 8.2	5 ± 4.5	-	UKPDSBBC	-	-	-
**Zago 2022**	**110**	Italy/Germany	dnPD (61)	77	37	41	66 ± 8.2	66.5 ± 8	-	-	-	sporadic idiopathic	Untreated	-
			dnPD (144); adPD (12)	35	dnPD (74); adPD (9)	16	dnPD (65 ± 12); adPD (68 ± 6)	75.5 ± 8.5	-	-	UKPDSBBC		Untreated (dnPD); Treated (adPD)	-
**Zhang 2017**	**44**	China	46	49	22	22	63.1 ± 1.5	60.4 ± 1.2	-	1–5	UKPDSBBC	Sporadic	-	-
**Zhang 2020**	**81**	China	148	126	86	74	71.4 ± 3.6	65.5 ± 3.4	-	1–5	UKPDSBBC	-	-	-

**Zhao 2014**	**82**	China	46	46	25	22	60.2 ± 1.4	61.4 ± 1.3	-	1–5	UKPDSBBC	Tremor-dominant, bradykinesia rigidity-dominant, mixed	-	-
**Zhou 2016**	**83**	China	12	12	6	6	63 ~ 78-year	63 ~ 78-year	-	-	-	-	Untreated	-
**Zhu 2021**	**84**	China	10	5	-	-	-	-	-	-	-	-	-	-

Supplementary Table S2. MicroRNA expressions reported to be significantly changed between Parkinson's disease patients and controls in the literature. [a]*de-novo* Parkinson's disease patients; [b] early-onset Parkinson's disease patients on dopaminergic therapy; **upward trend only, no statistical significance. DA – dopamine; HC – healthy controls; iPSC – induced pluripotent stem cells; PD – Parkinson's disease; PBLs – peripheral blood lymphocytes; PBMCs – peripheral blood mononuclear cells; SN – substantia nigra; CSF – cerebrospinal fluid; NGS – next generation sequencing; RNase – ribonuclease; RT-qPCR – quantitative reverse transcription polymerase chain reaction; TLDA – TaqMan Low-Density Array.

Study	Reference	Country	sample size	# PD	# HC	Sample	Method of MiRNA Detection	Normalizer	Up	Down	AUC
**Alieva 2015**	**124**	Russia	62	38	24	PBLs	RT-qPCR (TaqMan)	U6, U44, U47 snRNA	miR-7-5p, miR-9-3p, miR-9-5p, miR-129-5p, miR-132-3p	-	-
**Alvarez-Erviti 2013**	**94**	Spain	11	6	5	Brain (SN, amygdala)	RT-qPCR (TaqMan)	-	miR-21-3p, miR-26b-5p, miR-106a-3p, miR-224-5p, miR-301b-3p, miR-373-5p	-	-
**Baghi 2020**	**115**	Iran	58	33	25	PBMCs	RT-qPCR (SYBR Green ExiLENT)	U6 snRNA	miR-376a-3p	-	miR-376a-3p (0.80)
**Baghi 2021**	**116**	Iran	40	20	20	PBMCs	RT-qPCR (SYBR Green)	U6 snRNA	miR-193b	-	miR-193b (0.79)
**Bai 2017**	**58**	China	160	80	80	Serum	RT-qPCR (miRcute)	cel-miR-39	-	miR-29a-3p, miR-29b-3p, miR-29c-3p	-
**Barbagallo 2020**	**29**	Italy	60	30	30	Serum	RT-qPCR (TaqMan)	miR-17-5p	let-7d-5p, miR-22-5p, miR-23a-3p, miR-24-3p, miR-142-3p, miR-222-3p	-	let-7d (0.75), miR-22* (0.85), miR-23a (0.87), miR-24 (0.78), miR-142-3p (0.78), miR-222 (0.82), combined (0.94)
**Behbahanipour 2019**	**117**	Iran	52	36	16	PBMCs	RT-qPCR (SYBR Green)	miR-191-5p	miR-17-5p, miR-885-5p	miR-361-5p	miR-17-5p (0.76), miR-885-5p (0.81), miR-361-5p (0.76);

Study	Reference	Country	sample size	# PD	# HC	Sample	Method of MiRNA Detection	Normalizer	Up	Down	AUC
											combined (0.99)
**Bissonnette 2018**	**85**	USA	20	10	10	CSF	NGS (HTG EdgeSeq)	-	miR-578, miR-766-5p, miR-889-3p, miR-4736	-	-
**Botta-Orfila 2014**	**30**	Spain	130	65	65	Serum	TLDA, RT-qPCR (TaqMan)	miR-17-5p, miR-106	-	miR-19a-3p, miR-19b-3p, miR-29a-3p, miR-29c-3p	miR-19a-3p, miR-19b-3p, miR-29a-3p, miR-29c-3p (0.77)
**Briggs 2015**	**86**	USA	16	8	8	Brain (SN)	TLDA	Global mean	let-7b-5p, miR-26a-5p, miR-28-5p, miR-92a-3p, miR-95-3p, miR-106a-5p, miR-135a-5p, miR-145-5p, miR-148a-3p, miR-223-3p, miR-335-3p, miR-374a-5p	miR-532-5p, miR-744-5p	-
**Burgos 2014**	**87**	USA	112	50	62	Serum	NGS (Illumina HiSeq2000)	DESeq2	miR-30a-3p, miR-30e-3p, miR-338-3p	miR-16-2-3p, miR-1294	-
			122	57	65	CSF			miR-19a-3p, miR-19b-3p, let-7g-3p	miR-10a-5p, miR-127-3p, miR-128, miR-132-5p, miR-136-3p, miR-212-3p, miR-370, miR-409-3p, miR-431-3p, miR-433-3p, miR-485-5p, miR-873-3p, miR-1224-5p, miR-4448	-
**Caggiu 2018**	**103**	Italy	80	37	43	PBMCs	RT-qPCR (Qiagen)	U6 snRNA, SNORD68 snoRNA	miR-155-5p	miR-146a-5p	-
**Caldi Gomes 2022**	**111**	Germany	31	19	12	Midbrain	NGS (Illumina's HiSeq4000)	DESeq2	miR-539-3p, miR-376a-5p, miR-218-5p, miR-369-3p	-	-

Study	Reference	Country	sample size	# PD	# HC	Sample	Method of MiRNA Detection	Normalizer	Up	Down	AUC
**Cao 2017**	**31**	China	149	109	40	Serum	RT-qPCR (SYBR, Takara)	let-7d, let-7g, let-7i, cel-miR-39	miR-24-3p, miR-195-5p	miR-19b-3p	miR-19b (0.75), miR-24 (0.91), miR-195 (0.70); combined (0.95)
**Cardo 2013**	**95**	Spain	56	31	25	Plasma	TLDA, RT-qPCR (TaqMan)	miR-191	miR-331-5p	-	-
**Cardo 2014**	**96**	Spain	12	8	4	Brain (SN, striatum)	TLDA, RT-qPCR (TaqMan)	U6, U44 snRNA	miR-548d-3p	miR-135b-5p, miR-198, miR-208b-3p, miR-299-5p, miR-330-5p, miR-337-5p, miR-339-5p, miR-379-5p, miR-485-5p, miR-542-3p	-
**Chen 2016**	**59**	China	85	24	61	PBMCs	Microarray (GeneChip miRNA 3.0 Array, Affymetrix),RT-qPCR (SYBR Green)	RNU6	-	miR-451, miR-3935	-
**Chen 2017**	**45**	China	339	169	170	Plasma	Microarray (miRCURY LNA array v.18, Exiqon), RT-qPCR	miR-191-5p	miR-4639-5p	-	miR-4639-5p (0.94)
**Chen 2018**	**60**	China	50	25	25	Plasma	RT-qPCR (SYBR Green, Applied Biosystems)	5s rRNA	miR-27a-3p	let-7a-5p, let-7f-5p, miR-21-5p, miR-30a-5p, miR-125b-5p, miR-130a-3p, miR-130b-3p, miR-142-3p, miR-185-5p, miR-200a-3p, miR-222-3p, miR-423-5p, miR-485-5p, miR-874	let-7a-5p (0.90); let-7f-5p (0.85); miR-21-5p, (0.62); miR-27a-3p, (0.80); miR-30a-5p, (0.70); miR-125b-5p, (0.66); miR-130a-3p, (0.57); miR-130b-3p, (0.65); miR-142-3p, (0.96); miR-185-5p, (0.53); miR-

Study	Reference	Country	sample size	# PD	# HC	Sample	Method of MiRNA Detection	Normalizer	Up	Down	AUC
											200a-3p, (0.66);, miR-222-3p, (0.83); miR-423-5p, (0.66); miR-485-5p, (0.6); miR-874, (0.65)
**Chen 2020**	**46**	China	60	30	30	Saliva	RT-qPCR (TaqMan)	miR-16	miR-874, miR-145-3p	-	miR-874 (0.73); miR-145-3p (0.71)
**Chen 2021**	**32**	China	91	46	45	plasma	RT-qPCR	cel-miR-54-5p, hsa-miR-25-3p	miR-133b, miR-221-3p	-	miR-133b (0.84); miR-221-3p (0.64); miR-133b, miR-221-3p (0.85)
**Chis 2021**	**132**	Romania	95	66	29	plasma	RT-qPCR (TaqMan)	miR-15b, miR-17, cel-miR-39	miR-16, miR-19a, miR-19b, miR-92a, miR-195	-	-
**Chiu 2019**	**126**	Taiwan	100	50	50	Serum	RT-qPCR (Qiagen)	U6 snRNA	miR-204-5p	miR-26a-5p, miR-138-5p, miR-218-5p	-
**Cho 2013**	**88**	USA	26	15	11	Brain (SN)	RT-qPCR (miRCURY SYBR Green, Exiqon and Qiagen)	U6 snRNA	-	miR-205-5p	-
			9	5	4	Brain (striatum)				miR-205-5p	-
**Cosín-Tomás 2017**	**97**	Spain	41	20	21	Plasma	RT-qPCR (TaqMan)	miR-17-5p, miR-106a-5p, cel-miR-39	miR-15b-5p, miR-142-3p	-	-
**Cressatti 2020**	**47**	Canada	166	84	82	Saliva	RT-qPCR (SensiFast SYBR Lo-ROX, Frogga Bio)	U44 snRNA	-	miR-153, miR-223-5p	miR-153 (0.79); miR-223-5p (0.74)

Study	Reference	Country	sample size	# PD	# HC	Sample	Method of MiRNA Detection	Normalizer	Up	Down	AUC
**Ding 2016**	**33**	China	116	61	55	Serum	NGS (Solexa), RT-qPCR (TaqMan)	let-7d, let-7g, let-7i	miR-195-5p	miR-15b-5p, miR-181a-5p, miR-185-5p, miR-221-3p	miR-15b-5p (0.9); miR-181a-5p (0.82); miR-185-5p (0.82); miR-195-5p (0.75); miR-221-3p (0.85); miR-15b-5p, miR-181a-5p, miR-185-5p, miR-195-5p, miR-221-3p (0.92)
**Dong 2016**	**34**	China	166	92	74	Serum	NGS (Solexa), RT-qPCR (TaqMan)	Serum volume	-	miR-141-3p, miR-146b-5p, miR-193a-3p, miR-214-3p	miR-141 (0.85); miR-214 (0.89); miR-146b-5p (0.86); miR-193a-3p (0.82); combined (0.84)
**Dos Santos 2018**	**35**	Germany	80	40	40	CSF exosomes	NGS	DESeq2	-	-	let-7f-5p, miR-27a- 3p, miR-125a-5p, miR-151a-3p, miR-423-5p (0.82)
**Fazeli 2020**	**36**	Iran	44	30	14	PBMCs	RT-qPCR (SYBR Green)	-	miR-27b-3p	miR-27a-3p	miR-27a-3p (0.89), miR-27b-3p (0.67), miR-27a-3p/miR-27b-3p (0.81)
**Fernández-Santiago 2015**	**98**	Spain	36	8	28	Serum	RT-qPCR (TaqMan)	miR-17-5p, miR-106	-	miR-19b-3p, miR-29c-3p	-
**Fu 2017**	**9**	China	30	15	15	PBMCs	RT-qPCR (TaqMan)	U6 snRNA	miR-21-5p	-	-

Study	Reference	Country	sample size	# PD	# HC	Sample	Method of MiRNA Detection	Normalizer	Up	Down	AUC
**Ghit 2022**	**128**	Egypt	35	20	15	Serum	RT-qPCR (SYBR Green)	miRNeasy Serum/Plasma a SpikeIn control	-	miR-214, miR-221, miR-141	-
**Gong 2022**	**61**	China	10	5	5	Midbrain	RT-qPCR (SYBR Green)	U6 snRNA	miR-132-3p	-	-
**Grossi 2021**	**104**	Italy	29	15	14	Plasma SEV	RT-qPCR (TaqMan)	cel-miR-39, has-miR-23b-3p	miR-34a-5p	-	miR-34-5p (0.74)
**Gui 2015**	**62**	China	113	78	35	CSF exosomes	TLDA, RT-qPCR (TaqMan)	U6 snRNA	miR-10a-5p, miR-153-3p, miR-409-3p, let-7g-3p, miR-433, miR-136-3p	miR-1-3p, miR-19b-3p	miR-1-3p (0.92); miR-10a-5p (0.90); miR-19b-3p (0.71); miR-153-5p (0.78); miR-409-3p (0.97)
**Han 2020**	**63**	China	79	39	40	Serum	RT-qPCR	miRNeasy Serum Spike-In Control	-	-	-
**He 2021**	**64**	China	50	7 stage II; 12 stage III; 11 stage IV	10	Serum EV	RT-qPCR (SYBR Green)	U6 snRNA	miR-374a-5p, miR-374b-5p; stage II, III and IV; miR-28-5p, stage III; miR-22-5p, stage IV	miR-199a-3p, miR-195-5p; stage II; miR-151a-5p, stage IV	miR-374a-5p (0.76, 0.78, 0.76), miR-374b-5p (0.80, 0.74, 0.76); stage II, III and IV; miR-28-5p (0.79); stage III; miR-22-5p (0.70); stage IV; miR-199a-3p (0.74); stage II; miR-151a-5p (0.71); stage IV

**Study**	**Reference**	**Country**	**sample size**	**# PD**	**# HC**	**Sample**	**Method of MiRNA Detection**	**Normalizer**	**Up**	**Down**	**AUC**
**Hoss 2016**	**4**	USA	62	29	33	Brain (prefrontal cortex Brodmann Area 9)	NGS (Illumina HiSeq 2000)	DESeq2	miR-15a-5p, miR-15b-5p, miR-16-3p, miR-16-5p, miR-17-3p, miR-29a-3p, miR-92b-3p, miR-95-3p, miR-106a-5p, miR-130b-5p, miR-142-5p, miR-144-3p, miR-144-5p, miR-148b-5p, miR-151b, miR-181d-3p, miR-208b-3p, miR-210-5p, miR-216b-5p, miR-330-5p, miR-340-3p, miR-361-5p, miR-363-3p, miR-369-5p, miR-376c-5p, miR-380-3p, miR-381-3p, miR-383-5p, miR-411-5p, miR-421, miR-423-3p, miR-424-3p, miR-450a-5p, miR-487a-3p, miR-516a-5p, miR-516b-5p, miR-519a-3p, miR-522-3p, miR-539-3p, miR-545-5p, miR-548o-3p, miR-576-5p, miR-589-5p, miR-628-3p, miR-628-5p, miR-654-3p, miR-656-3p, miR-874-5p, miR-1306-3p, miR-1468-5p, miR-3117-3p, miR-3127-5p, miR-3660, miR-3909, miR-3912-3p, miR-4443, miR-4526, miR-5100, miR-5690, miR-5701, miR-6841-5p	miR-10b-5p, miR-26a-1-3p, miR-29b-2-5p, miR-29c-5p, miR-30a-3p, miR-30c-2-3p, miR-103a-2-5p, miR-124-5p, miR-127-3p, miR-129-1-3p, miR-129-2-3p, miR-129-5p, miR-132-3p, miR-132-5p, miR-134-5p, miR-138-2-3p, miR-145-5p, miR-148b-3p, miR-152-3p, miR-154-5p, miR-184, miR-185-3p, miR-212-3p, miR-212-5p, miR-217, miR-320b, miR-323a-5p, miR-328-3p, miR-329-3p, miR-331-3p, miR-338-5p, miR-362-5p, miR-376c-3p, miR-377-5p, miR-378c, miR-380-5p, miR-382-3p, miR-382-5p, miR-425-3p, miR-432-5p, miR-433-3p, miR-490-5p, miR-491-5p, miR-501-5p, miR-548w, miR-655-3p, miR-670-3p, miR-671-5p, miR-937-3p, miR-1224-5p, miR-1262, miR-1294, miR-2277-3p, miR-3200-3p, miR-3928-3p, miR-3943, miR-4705, miR-4787-3p, miR-6511a-5p, miR-6810-3p, miR-6864-5p, miR-6865-3p, let-7i-3p, let-7i-5p	-

Study	Reference	Country	sample size	# PD	# HC	Sample	Method of MiRNA Detection	Normalizer	Up	Down	AUC
**Jiang 2021**	**37**	China	80	50	30	Saliva	Agilent Human miRNA Microarray, RT-qPCR	U6 snRNA	miR-6756-5p	miR-29a-3p, miR-29c-3p	miR-29a-3p (0.69); miR-29c-3p (0.72); miR-6756-5p (0.64); miR-29a-3p + miR-29c-3p (0.77)
**Jin 2018**	**10**	China	92	46	46	Serum	RT-qPCR (TaqMan)	U6 snRNA	miR-520d-5p	-	-
**Khoo 2012**	**48**	USA	72	42	30	Plasma	Microarray (Agilent v.3), RT-qPCR (TaqMan)	miR-16, ath-miR-156a	miR-626, miR-505-3p, miR-222	-	miR-626 (0.93); miR-505-3p (0.89); miR-222 (0.79)
**Kim 2007**	**89**	USA	6	3	3	Brain (midbrain)	RT-qPCR (Qiagen)	-	-	miR-133b	-
**Kurz 2021**	**112**	Germany	30	13	17	sigmoid colon	NGS (Illumina High-Throughput Sequencing)	DESeq2	miR-486–5p	-	miR-486-5p (0.73)
**Li 2017**	**49**	China	120	60	60	Plasma	RT-qPCR (Mir-X SYBR Kit, TaKaRa)	U6 snRNA	miR-137	miR-124-3p	miR-137 (0.71); miR-124 (0.71)
**Li 2020**	**50**	China	140	80	60	Serum	RT-qPCR (SYBR Green)	U6 snRNA	-	miR-150-5p	miR-150-5p (0.88)
**Li 2021**	**51**	China	90	69	21	Serum	RT-qPCR (SYBR Green)	External reference	miR-31 (aPD); miR-214 (pPD)	-	miR-31 (aPD= 0.74); miR-214 (pPD=0.76)
**Lin 2022**	**65**	China	158	92	64	Serum	RT-qPCR (SYBR Green)	U6 snRNA	miR-485-3p	-	miR-485-3p (0.92)
**Ma 2016**	**66**	China	250	138	112	Serum	RT-qPCR (TaqMan)	let-7d/g/i	-	miR-29c-3p, miR-146a-5p, miR-214-3p, miR-221-3p	miR-221-3p (0.79); miR-29c-3p (0.67); miR-146a-5p (0.66); miR-214-3p (0.79)
**Manna 2021**	**38**	Italy	84	40	39	Serum exosomes	RT-qPCR (TaqMan)	global median	miR-22-3p, miR-223-5p	miR-21-3p	miR-22-3p, miR-223-5p, miR-21-3p (0.75)

Study	Reference	Country	sample size	# PD	# HC	Sample	Method of MiRNA Detection	Normalizer	Up	Down	AUC
**Margis 2011**	**8**	Brazil	16	8	8	Whole blood	Microarray, RT-qPCR (SYBR Green)	-	-	miR-1-3p, miR-29a-3p	-
			15	7	8			-	miR-16-2-3p, miR-26a-2-3p	-	-
**Marques 2017**	**39**	The Netherlands	56	28	28	CSF	RT-qPCR (TaqMan)	miR-16-5p, U6 snRNA	miR-205-5p	miR-24-3p	miR-205-5p (0.73); miR-24-3p (0.71); miR-19a, miR-19b, miR-24, miR-30c, miR-34b, miR-133b, miR-205 (0.96)
**Martins 2011**	**121**	Portugal	32	19	13	PBMCs	Microarray (miRCURY LNA array v.10, Exiqon), RT-qPCR (TaqMan)	miR-103	miR-15b-5p, miR-550	miR-32-5p, miR-101-3p, miR-126-5p	-
**McMillan 2017**	**127**	UK	11	6	5	Brain (SN)	RT-qPCR (TaqMan)	U6, U58 snRNA	-	miR-7-5p	-
**Miñones-Moyano 2011**	**99**	Spain	44	16	28	Brain (amygdala, SN, frontal cortex, cerebellum)	Microarray (miRCURYLNA array, Exiqon), RT-qPCR (TaqMan)	U6 snRNA	-	miR-34b-3p, miR-34c-5p	-
**Mo 2017**	**53**	China	86	44	42	CSF	RT-qPCR (TaqMan)	U6 snRNA	miR-144-5p, miR-200a-3p, miR-542-3p	-	miR-144-5p (0.73); miR-200a-3p (0.75); miR-542-3p (0.87)
**Nair 2016**	**90**	USA	24	12	12	Brain (putamen)	miRNA assay (nCounter Human v2, Nanostring Technologies), RT-qPCR (TaqMan)	Global mean	miR-95-3p, miR-204-5p, miR-221-3p, miR-425-5p, miR-485-3p, miR-3195	miR-155-5p, miR-219-2-3p, miR-382-5p, miR-421, miR-423-5p, miR-3200-3p, miR-4421	-

Study	Reference	Country	sample size	# PD	# HC	Sample	Method of MiRNA Detection	Normalizer	Up	Down	AUC
**Nie 2020**	**67**	China	41	7	34	Plasma exosomes	NGS (BGISEQ-500)	DESeq2	miR-378g, miR-652-3p, miR-3184-3p, miR-4732-3p, miR-6131, let-7e-5p, let-7i-5p	miR-30d-5p, miR-93-5p, miR-99b-5p, miR-122-3p, miR-192-5p, miR-197-3p, miR-211-5p, miR-375, miR-425-5p, miR-576-5p, miR-941 miR-1468-5p, let-7e-3p	-
**Oliveira 2020**	**122**	Portugal	80	40	40	Serum	RT-qPCR (TaqMan)	cel-miR-39-3p	-	miR-146a, miR-335-3p, miR-335-5p	miR-146a (0.68), miR-335-3p (0.70), miR-335-5p (0.66); combined all 3 miRNAs (0.72)
**Ozdilek 2020**	**54**	Turkey	71	51	20	Serum	RT-qPCR (SYBR Green)	miR-191, miR-1228, cel-miR-39	miR-29c-3p	-	miR-29c -3p (0.69)
**Patil 2019**	**91**	USA/ Norway	64	42	22	Serum	Microarray (Affimetrix GeneChip miRNA 4.0),RT-qPCR (Quanta Biosciences)	scaRNA17, cel-miR-39-3p	miR-335-5p, miR-3613-3p, miR-6865-3p		miR-335-5p/miR-3613-3p (0.75), miR-3613-3p/miR-6865-3p (0.75), miR-335-5p/miR-6865-3p (0.71) and miR-335-5p/miR-3613-3p/miR-6865-3p (0.76)
**Perez-Soriano 2020**	**100**	Spain	38	18	20	Serum	RT-qPCR (TaqMan)	miR-320a-3p, miR-6727-5p	-	-	-
**Qin 2019**	**68**	China	44	20	24	CSF	RT-qPCR (TaqMan)	miR-24	-	miR-626	-

Study	Reference	Country	sample size	# PD	# HC	Sample	Method of MiRNA Detection	Normalizer	Up	Down	AUC
**Ravanidis 2019; 2020**	**41**	Greece	401	208	193	Plasma	RT-qPCR (SYBR)	miR-103α-3p, miR-16-5p, miR-191-5p, miR-223-3p, miR-425-5p, miR-423-3p	miR-22-3p, miR-124-3p, miR-136-3p, miR-154-5p, miR-323a-3p	-	Pooled study (0.74); miR-7-5p, miR-136-3p, miR-409-3p
**Schlaudraff 2014**	**113**	Germany	13	5	8	Brain (midbrain)	RT-qPCR (Qiagen)	β-actin, TIF-1A, ENO2	-	miR-133b	-
**Schwienbacher 2017**	**105**	Italy	198	100	98	Plasma, WBC	RT-qPCR (TaqMan)	cel-miR-39-3p, cel-miR-54-3p, cel-miR-238-3p	-	-	-
			20	10	10				-	-	-
**Serafin 2015**	**106**	Italy	72	36	36	PBMCs	RT-qPCR (TaqMan)	miR-103a-3p, U6 snRNA, Z30, RNU24 snoRNAs	miR-29a-3p, miR-30b-5p, miR-103a-3p	-	-
**Sethi 2009**	**92**	USA	10	4	6	Brain (temporal lobe neocortex Brodmann Area A22)	Microarray (LC Sciences)	5S RNA	-	-	-
**Sheinerman 2017**	**42**	USA	100	50	50	Plasma	RT-qPCR (TaqMan)	miR-16, miR-27a	–	–	miR-9-3p/miR-129-3p (0.91); miR-127-3p/miR-411-5p (0.81); miR-491-5p/miR-138-5p (0.81)
**Shu 2020**	**69**	China	126	82	44	Serum	RT-qPCR	U6 snRNA	-	miR-132-3p, miR-146a-5p	miR-132-3p (0.73); miR-146a-5p (0.73)

Study	Reference	Country	sample size	# PD	# HC	Sample	Method of MiRNA Detection	Normalizer	Up	Down	AUC
**Soreq 2013**	**56**	Israel	13	7	6	Peripheral blood leukocytes	NGS (SOLid system V3.5, Applied Biosystems)	Quantile normalization	miR-18b-3p, miR-21-5p, miR-20a-5p, miR-150-5p, miR-199b-5p, miR-378c, miR-671-5p, miR-1249, miR-1274b, miR-4293	miR-16-5p, miR-92b-3p, miR-320a, miR-320b, miR-320c, miR-769	-
**Starhof 2018**	**125**	Denmark	60	37	23	CSF	Microarray (miRCURY v.4, Exiqon), RT-qPCR (TaqMan)	Global mean	miR-7-5p, miR-34c-3p, miR-106b-5p, miR-184, miR-218-5p, miR-331-5p	miR-99a-5p, let-7b-5p	miR-7-5p + miR-331-5p + miR-145-5p (0.88)
						Plasma			miR-30c-5p, miR-191-5p, miR-218-5p, miR-873-3p	miR-574-3p	-
**Su 2019**	**70**	China	56	28	28	CSF	RT-qPCR (Ribo Bio)	U6 snRNA	-	miR-26a-5p	-
**Sulaiman 2020**	**129**	Malaysia	39	28	11	Plasma	Affymetrix GeneChip miRNA v4.0	R software via the Affy package	miR-301a-3p, miR-100-5p, miR-140-5p, miR-486-3p, miR-143-3p	-	-
**Takahashi 2015**	**119**	Japan	77	30	47	Plasma	miRNA oligo chip (TORAY Industries, v.17), RT-qPCR (SYBR Green, Qiagen)	miR-4516	miR-663b	-	-

Study	Reference	Country	sample size	# PD	# HC	Sample	Method of MiRNA Detection	Normalizer	Up	Down	AUC
**Tan 2022**	**71**	China	11	7	4	CSF	NGS (Hiseq2500)	DESeq2	miR-486-5p, miR-122-5p, miR-451a, miR-423-5p , let-7b-5p, miR-151a-3p, miR-320a, miR-574-5p, miR-206, miR-204-5p, miR-1298-5p, miR-320b, miR-1246, miR-1307-3p, miR-128-3p, miR-409-3p, let-7a-5p, miR-144-3p, let-7d-3p, miR-4508, miR-155-5p	-	-
**Tatura 2016**	**114**	Germany	32	22	10	Brain (anterior cingulate gyrus)	TLDA, RT-qPCR (SYBR Green)	U6 snRNA	miR-144-3p, miR-199b-5p, miR-221-3p, miR-488-3p, miR-544	-	-
**Thomas 2012**	**93**	USA	16	8	8	Brain (frontal cortex)	Microarray (Agilent)	-	miR-144-3p, miR-148a-3p, miR-190, miR-195-3p, miR-199a-3p, miR-200a-3p, miR-200a-5p, miR-200b-5p, miR-424-3p, miR-429, miR-451	-	-
**Tolosa 2018**	**101**	Spain	10	6	4	iPSC-derived DA neurons	TLDA, RT-qPCR (TaqMan, SYBR Green)	U6 snRNA, RNU48 snoRNA, miR-26a, miR-484, miR-744, miR-26b	miR-9-5p, miR-135a-5p, miR-135b-5p, miR-449a, miR-449b-5p	miR-141-3p, miR-199a-5p, miR-299-5p, miR-518e-3p, miR-519a-3p	-
**Tong 2022**	**72**	China	259	209	50	Serum exosomes	TLDA, RT-qPCR (TaqMan)	RNU6-B	miR-151a-5p, miR-24, mir-485-5p, mir-331-5p, mir-214	-	miR-151a-5p (0.86); miR-24 (0.88); miR-331-5p (0.88); miR-

Study	Reference	Country	sample size	# PD	# HC	Sample	Method of MiRNA Detection	Normalizer	Up	Down	AUC
						CSF exosomes					485-5p (0.90) ; miR-214 (0.93)
**Uwatoko 2019**	**120**	Japan	56	28	28	Plasma	Microarray (RT-qPCR (SYBR Green, Qiagen)	Global mean (microarray); miR-4516 (RT-qPCR)	miR-19b-3p	miR-671-5p	-
**Vallelunga 2014**	**107**	Italy	50	25	25	Serum	TLDA, RT-qPCR (TaqMan)	Global mean (TLDA) miR-17, miR-151-3p (RT-qPCR)	miR-24-3p, miR-223-5p, miR-324-3p	miR-30c-5p, miR-148b-3p	-
**Vallelunga 2019**	**108**	Italy	106	56	50	Serum	RT-qPCR (miRCURY LNA assay, Exiqon)	miR-93-5p	miR-30c-5p	-	-
**Vallelunga 2021**	**109**	Italy	107	51	56	Serum	RT-qPCR (Exiqon)	miR-93-5p	-	miR-96-5p	miR-96-5p (0.64)
**Villar-Menéndez 2014**	**102**	Spain	34	25	9	Brain (putamen)	RT-qPCR (Exiqon)	RD38B snoRNA	-	miR-34b-3p	-
**Wake 2016**	**57**	USA	65	29	36	Brain (prefrontal cortex Brodmann 9)	NGS (Illumina HiSeq 2000), RT-qPCR (miRCURY LNA assay, Exiqon)	-	-	-	-
**Wang 2020**	**73**	China	240	120	120	Plasma	RT-PCR (SYBR Green)	U6 snRNA	-	miR-103, miR-107	-
**Wu 2020**	**43**	China	135	85	50	Serum	RT-PCR (SYBR Green)	U6 snRNA	-	miR-9a, miR-133b	miR-9a (0.90); miR-133b (0.89); combined (0.96)
**Xing 2020**	**74**	China	25	15	10	Brain (prefrontal cortex)	RT-qPCR (TaqMan)	U6 snRNA	-	miR-124-3p, miR-144-3p, miR-218-5p	-

Study	Reference	Country	sample size	# PD	# HC	Sample	Method of MiRNA Detection	Normalizer	Up	Down	AUC
**Yan 2020**	**75**	China	47	23	24	Serum	RT-qPCR (TaqMan)	to the level of the external reference	miR-214	-	miR-214 (0.71)
**Yang 2018**	**76**	China	60	30	30	Serum exosomes	RT-qPCR (Sangon Biotech)	U6 snRNA	miR-135a-5p	-	-
**Yang 2019 A**	**77**	China	592	319	273	Plasma	RT-qPCR (Tiangen Biotech)	miR-16	miR-105-5p	-	miR-105-5p (0.77)
**Yang 2019 B**	**78**	China	491	269	222	Plasma	RT-qPCR (Tiangen Biotech)	artificial synthetic external miRNA	miR-132-3p	-	miR-132-3p (0.72)
**Yang 2021**	**79**	China	156	78	78	Peripheral blood	RT-PCR (SYBR Green)	U6 snRNA	-	miR-34a; miR-125a	miR-34a (0.81); miR-125a (0.78)
**Yao 2018**	**80**	China	185	52	48	Plasma exosomes	RT-qPCR (SYBR Green, Exiqon)	Endogenous control	miR-331-5p	miR-505-3p	miR-331-5p (0.85); miR-505-3p (0.90)
**Yilmaz 2016**	**123**	Turkey	204	102	102	Whole blood	RT-PCR (SYBR Green, Qiagen)	miR-26b-5p	-	miR-335-3p, miR-561-3p, miR-579-3p	-
**Yousefi 2022**	**118**	Iran	58	38	20	PBMCs	RT-qPCR (SYBR Green)	U6 snRNA	-	miR-24-3p; miR-30c-5p	miR-24-3p (0.82); miR-30c-5p (0.71)
**Zago 2022**	**110**	Italy				Serum	RT-qPCR (TaqMan)	miR-16-5p, miR-93-5p, miR-186-5p	miR-150-5p (dnPD, adPD)	miR-144-3p (dnPD)	-
**Zhang 2017**	**44**	China	95	46	49	Plasma	RT-qPCR (RiboBio)	U6 snRNA	-	miR-133b, miR-433-3p	miR-133b, miR-433-3p (0.59)
**Zhang 2020**	**81**	China	274	148	126	Serum	RT-qPCR (Shanghai GenePharma)	U6 snRNA	miR-30c-5p, miR-373	-	-
**Zhao 2014**	**82**	China	92	46	46	Serum	RT-qPCR (TaqMan)	U6 snRNA	-	miR-133b	-

Study	Reference	Country	sample size	# PD	# HC	Sample	Method of MiRNA Detection	Normalizer	Up	Down	AUC
**Zhou 2016**	**83**	China	24	12	12	Serum	RT-qPCR (SYBR Green)	U6 snRNA	-	miR-7-5p	-
**Zhu 2021**	**84**	China	15	10	5	Serum	RT-qPCR	U6 snRNA	miR-15b-5p	-	-

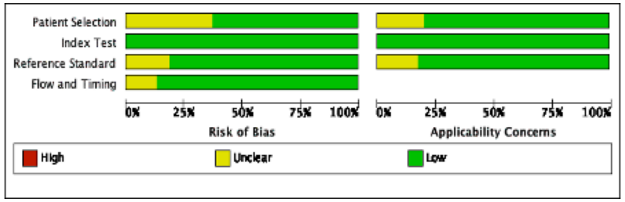

Supplementary Figure S1. Quality of studies included in the systematic review and meta-analysis using the QUADAS-2 quality assessment tool (n=102 studies).

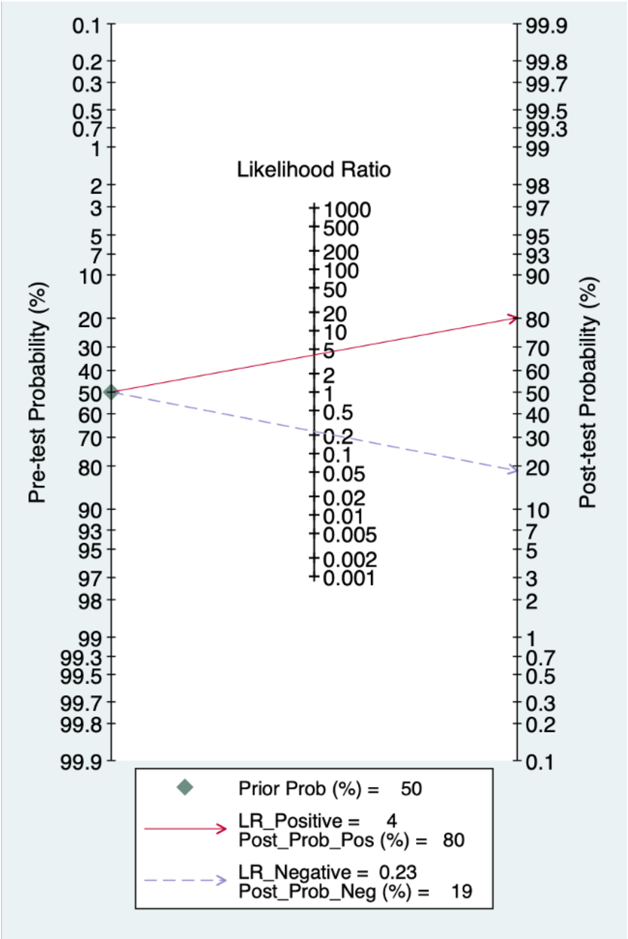

Supplementary Figure S2. Fagan's nomogram showing pre-test (left vertical axis) and post-test (right vertical axis) probabilities of biofluid-derived microRNAs able to discriminate between Parkinson's disease patients and controls with corresponding estimates for heterogeneity I2 tests (n=15 studies). The red arrow represents the positive likelihood ratio, and the purple dotted arrow represents the negative likelihood ratio.

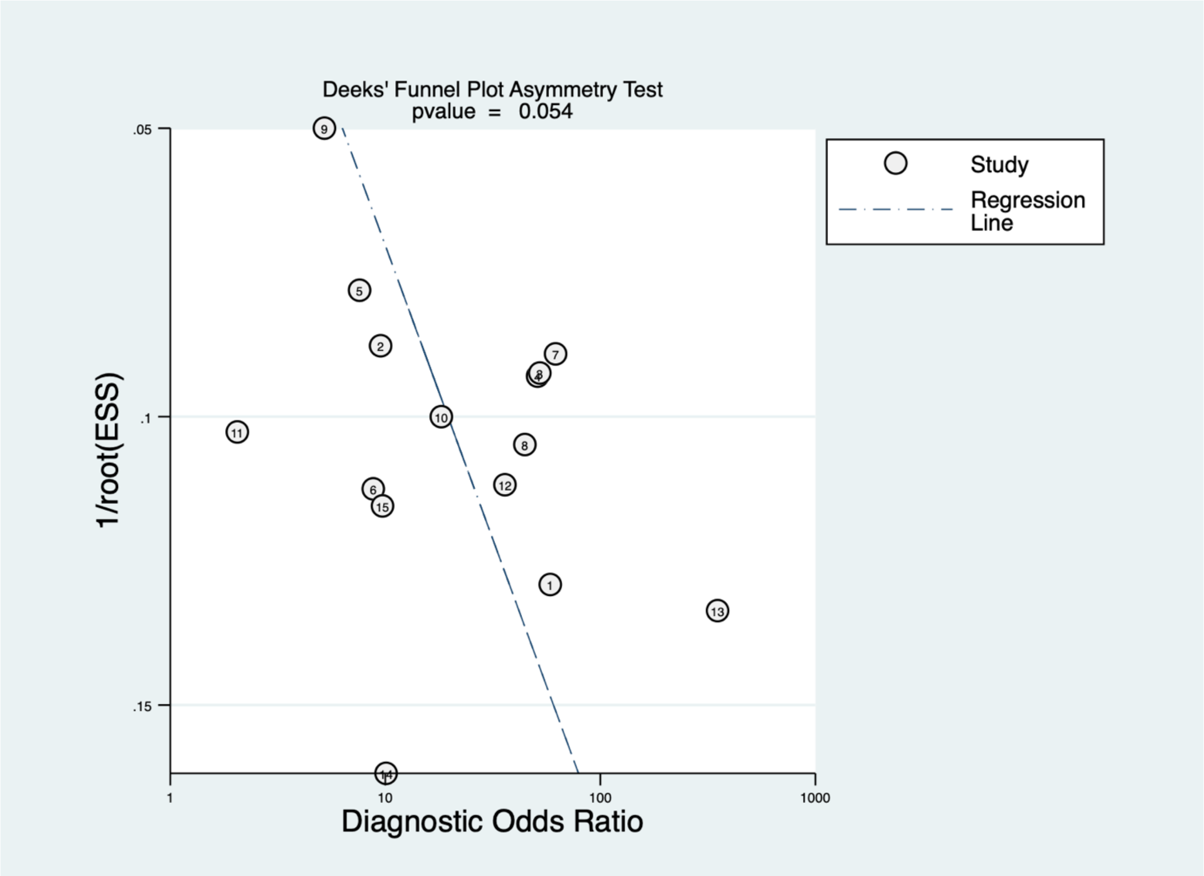

Supplementary Figure S3. Deeks' Funnel Plot Asymmetry Test with a superimposed regression line on a funnel plot indicating no significant publication bias in the analysis of biofluid-derived microRNAs from Parkinson's disease patients and controls (n=15 studies). The circles represent each study involved in the meta-analysis: 1= [[29]], 2= [[30]], 3= [[31]], 4= [[33]], 5= [[34]], 6= [[38]], 7= [[43]], 8= [[32]], 9= [[41]], 10= [[42]], 11= [[44]], 12= [[35]], 13= [[39]], 14= [[36]] and 15= [[37]].
